# Regulatory-prior-guided attention preserves biological structure during unpaired single-cell RNA–ATAC integration

**DOI:** 10.1093/bioinformatics/btag696

**Published:** 2026-09-18

**Authors:** Zhenglong Cheng, Jiao Zhang, Shixiong Zhang

**Affiliations:** School of Computer Science and Technology, Xidian University, Xi’an, Shaanxi 710126, China; Texas Children’s Cancer and Hematology Center, Texas Children’s Hospital, Houston, TX 77030, United States; Division of Hematology and Oncology, Department of Pediatrics, Baylor College of Medicine, Houston, TX 77030, United States; School of Computer Science and Technology, Xidian University, Xi’an, Shaanxi 710126, China

## Abstract

**Motivation:**

Single-cell RNA sequencing and single-cell ATAC sequencing provide complementary views of transcriptional output and chromatin regulatory potential, but integrating unpaired profiles remains challenging because the modalities differ in feature space, sparsity and noise. Existing approaches often frame integration as distribution matching, which can over-align biologically distinct, condition-specific or modality-specific cell states. We present scHPGT, a single-cell Heterogeneous Prior-Guided Transformer for regulatory-prior-guided integration of unpaired RNA and chromatin accessibility profiles. scHPGT uses modality-specific encoders to model RNA and ATAC signals, a prior-guided cross-modal Transformer to constrain gene–peak attention using regulatory links, and a domain-adversarial objective to reduce modality-specific discrepancies in a shared latent space.

**Results:**

Across PBMC3k, mouse spleen, CITE-seq/ASAP-seq PBMC and PBMC10k benchmarks, scHPGT improves clustering agreement, label transfer and biological structure preservation while maintaining effective modality alignment. In partial-overlap and condition-shift settings, scHPGT aligns shared populations without forcing unmatched or condition-specific states into inappropriate correspondence. Attention-derived links recover regulatory relationships, highlight marker-gene regulatory regions, recover transcription factor programs and produce regulatory activity profiles consistent with cell-type-specific transcriptional programs.

**Availability and Implementation:**

Code and datasets are released at https://github.com/zlCreator/scHPGT.

## 1 Introduction

Single-cell multi-omics technologies have transformed the study of cellular heterogeneity by enabling multiple molecular layers to be profiled in the same biological system (Ashuach *et al.* 2022). scRNA-seq measures transcriptional output, whereas scATAC-seq captures chromatin accessibility and regulatory potential. Integrating these modalities is essential for linking regulatory elements to gene expression programs and for constructing unified representations of cellular identity. This task is especially challenging in the unpaired setting, where RNA and ATAC profiles are measured from different cells, and no cell-to-cell correspondence is available.

The difficulty of unpaired integration arises from both statistical and biological sources. RNA and ATAC data differ in feature space, sparsity, dynamic range, and measurement noise. scATAC-seq profiles are high-dimensional and sparse, whereas scRNA-seq data are comparatively denser and gene-centric. Technical variation across platforms, batches, and preprocessing pipelines can further dominate shared biological signals. These factors produce a modality gap that can lead to under-alignment, where matching cell states remain separated by modality, or over-alignment, where biologically distinct states are inappropriately merged.

In the field of single-cell multi-omics and cross-condition integration, existing frameworks exhibit two prevalent shortcomings despite their contributions. The first is over-smoothing caused by aggressive alignment, a common issue in tools like GLUE (Cao and Gao 2022) and uniPort (Cao *et al.* 2022). Driven by strong domain adversarial loss or optimal transport, these methods frequently treat condition-induced biological shifts as non-interesting technical noise. Consequently, high nominal batch-mixing metrics are achieved at the expense of biological resolution, which can smooth away condition-specific activation responses and blur the boundaries between closely related lineages, ultimately compromising downstream differential analyses. The second issue concerns the limited representational capacity of highly heterogeneous feature spaces. Methods such as LIGER (Welch *et al.* 2019) (constrained by linear NMF), Cobolt (Gong *et al.* 2021) (lacking explicit prior-guided graph reasoning), and SCALEX (Xiong *et al.* 2022) (designed primarily for single-modality batch correction) struggle to capture high-order, non-linear peak-to-gene interactions. This limitation often prevents models from constructing a stable shared backbone across disparate multi-modal spaces.

To address these challenges, we developed scHPGT (single-cell Heterogeneous Prior-Guided Transformer), a framework that embeds peak-gene regulatory structure directly into cross-modal attention. scHPGT uses modality-specific encoders for RNA and ATAC profiles, a prior-guided Transformer that constrains attention to biologically plausible gene-peak relationships derived from promoter-proximal and peak-gene regulatory evidence (Pliner *et al.* 2018), and a domain-adversarial module that learns modality-invariant latent representations. This design decouples two requirements of robust integration: regulatory priors determine what information should be aligned, whereas adversarial learning determines how the modalities are aligned in a shared space.

We evaluate scHPGT across multiple unpaired RNA-ATAC integration tasks, including standard PBMC and mouse spleen benchmarks, unbalanced PBMC10k experiments, and cross-condition CITE-seq/ASAP-seq integration. Beyond benchmarking, we examine whether the model’s attention mechanism captures genuine regulatory information. Attention-derived links recover ground-truth gene-peak associations, high-attention peaks are enriched for functional cis-regulatory annotations, attention-selected peaks recover lineage-associated transcription factor programs, and attention-derived regulatory activity agrees with cell-type-specific transcriptional programs. These results support scHPGT as a biologically constrained and interpretable framework for unpaired single-cell multi-omics integration.

## 2 Methods

### 2.1 Overview of scHPGT

scHPGT integrates unpaired scRNA-seq and scATAC-seq profiles by combining heterogeneous feature encoding, prior-guided cross-modal attention, and domain-adversarial alignment (Fig. 1A–C). The model is motivated by the biological relationship between chromatin accessibility and transcriptional output: accessible promoters and enhancers provide regulatory potential for gene expression. Rather than relying only on statistical similarity between modalities, scHPGT uses peak-gene prior information to constrain cross-modal information flow.

**Figure 1:**
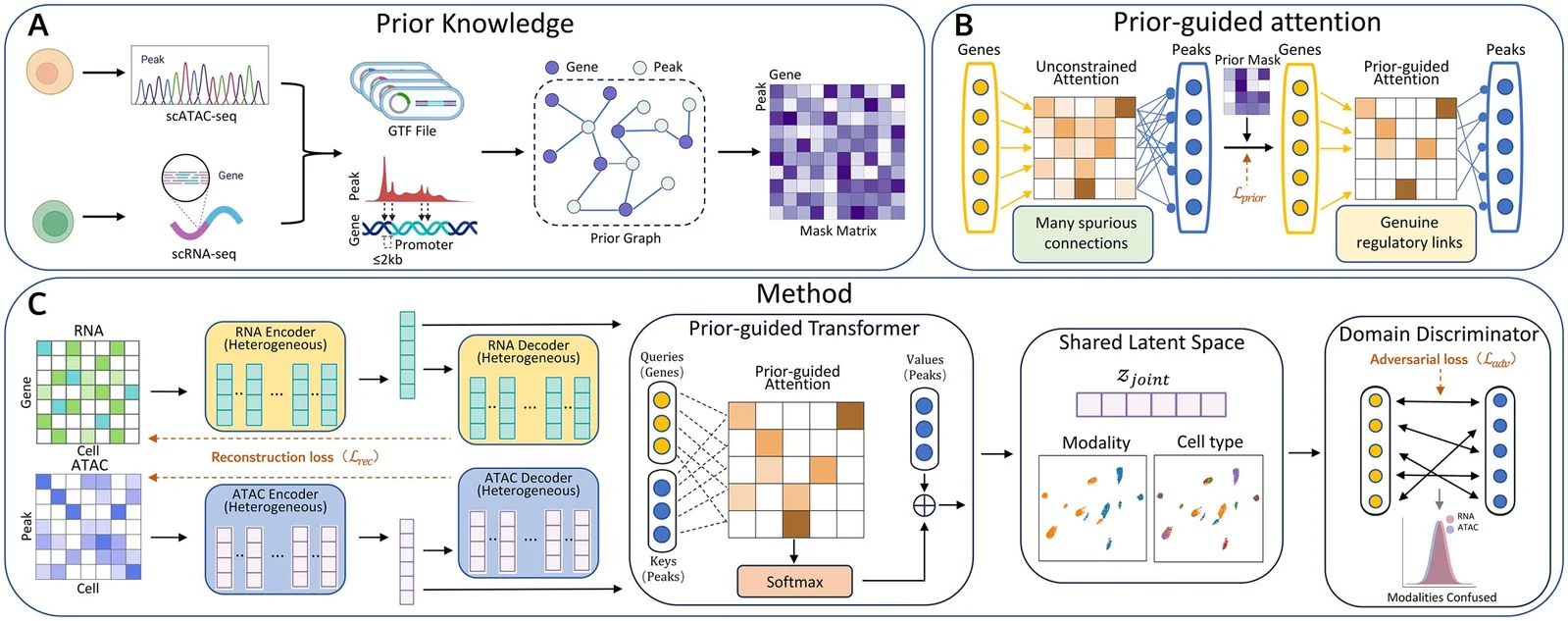
scHPGT performs biologically constrained integration of unpaired RNA and ATAC profiles. (A) Construction of a regulatory prior mask from genomic annotation and peak-gene relationships. (B) Prior masking transforms dense, weakly structured unconstrained attention into sparse attention enriched for genuine regulatory links. (C) scHPGT architecture. Heterogeneous RNA and ATAC encoders produce latent representations; the prior-guided Transformer performs masked cross-modal attention; a shared latent representation is optimized with reconstruction, prior-consistency, and adversarial objectives.

The framework has three components. First, modality-specific encoders learn latent representations for RNA and ATAC cells while preserving modality-specific signal through reconstruction (Fig. 1C). Second, a prior-guided Transformer uses genes as queries and peaks as keys and values, with attention restricted by a regulatory mask (Fig. 1B and C). Third, a domain discriminator with a gradient reversal layer reduces modality-specific information in the shared latent space (Fig. 1C).

### 2.2 Problem formulation

Let $X_{\mathrm{rna}}={\left\{x_{i}^{\mathrm{rna}}\right\}}_{i=1}^{N}$ and $X_{\mathrm{atac}}={\left\{x_{j}^{\mathrm{atac}}\right\}}_{j=1}^{M}$ denote the unpaired scRNA-seq and scATAC-seq datasets, where *N* and *M* are the number of cells. No one-to-one correspondence is assumed between cells across modalities. The objective is to learn modality-specific mappings into a shared latent space $Z\subset R^{d}$ such that biologically related cell states are close while modality-specific technical variation is minimized.

The feature spaces are not directly comparable: RNA profiles are defined over genes, whereas ATAC profiles are defined over genomic peaks. scHPGT addresses this mismatch by defining biologically plausible cross-modal interactions through a peak-gene prior graph and by using this graph to constrain attention.

### 2.3 Heterogeneous feature extraction

To account for modality-specific signal distributions, scHPGT uses independent RNA and ATAC encoders, $E_{\mathrm{rna}}$ and $E_{\mathrm{atac}}$. The encoders map each modality to a latent representation:


(1) $$z_{i}^{\mathrm{rna}}=E_{\mathrm{rna}}(x_{i}^{\mathrm{rna}}),\;z_{j}^{\mathrm{atac}}=E_{\mathrm{atac}}(x_{j}^{\mathrm{atac}})$$

In addition to cell-level embeddings, the heterogeneous encoders retain low-dimensional feature-token embeddings for genes and peaks. These feature embeddings are used by the prior-guided Transformer, whereas aggregated cell-level embeddings are used for reconstruction and domain-adversarial alignment.

Modality-specific decoders reconstruct the original inputs from the latent codes. The reconstruction loss preserves information and prevents the shared representation from collapsing during alignment:


(2) $$L_{\mathrm{rec}}=\sum_{i=1}^{N}∥x_{i}^{\mathrm{rna}}-{\hat{x}}_{i}^{\mathrm{rna}}{∥}^{2}+\sum_{j=1}^{M}∥x_{j}^{\mathrm{atac}}-{\hat{x}}_{j}^{\mathrm{atac}}{∥}^{2}$$

This reconstruction term counterbalances the alignment objectives and helps maintain biological heterogeneity within each modality.

### 2.4 Prior-guided cross-modal transformer

The prior-guided Transformer integrates RNA and ATAC latent representations through masked cross-modal attention. RNA and ATAC profiles are first processed by their modality-specific encoders. In addition to cell-level embeddings, the encoders produce low-dimensional gene and peak feature embeddings, denoted by $H^{\mathrm{gene}}\in R^{G\times d}$ and $H^{\mathrm{peak}}\in R^{P\times d}$, respectively. The Transformer performs cross-modal attention at the feature-token level: gene embeddings are used as queries, whereas peak embeddings are used as keys and values.


(3) $$Q=H^{\mathrm{gene}}W^{Q},\;K=H^{\mathrm{peak}}W^{K},\;V=H^{\mathrm{peak}}W^{V}$$

where $W^{Q},W^{K},W^{V}\in R^{d\times d}$ are learnable projection matrices. The regulatory prior is encoded as a sparse gene-by-peak matrix *M* in $R^{G\times P}$, where *G* denotes the number of genes and *P* denotes the number of ATAC peaks. An entry $M_{g,p}$ is non-zero when peak *p* is assigned to gene *g* by promoter proximity or by distal regulatory evidence, such as peak-gene links inferred from chromatin accessibility and gene-expression covariation. To reduce bias toward genes with many linked peaks, each row of *M* is normalized across the peaks linked to the same gene before it is used in attention. After obtaining *M*, we derive a binary support matrix $S_{g,p}=1(M_{g,p}>0)$ and convert it into an additive mask $B\in R^{G\times P}$, where $B_{g,p}=0$ if $S_{g,p}=1$ and $B_{g,p}=-\infty$ otherwise. Thus, *B* blocks unsupported gene-peak pairs in attention, while the normalized values in *M* are retained for the prior-consistency loss.

The masked attention operation is:


(4) $$M_{A}=\text{Softmax}\left(\frac{QK^{T}}{\sqrt{d}}+B\right),\;H^{\mathrm{cross}}=M_{A}V$$

where $M_{A}\in R^{G\times P}$ is the prior-guided gene-to-peak attention matrix and $H^{\mathrm{cross}}\in R^{G\times d}$ is the peak-informed gene representation. This operation can be interpreted as a structured sparsity constraint over cross-modal interactions. By suppressing attention on biologically unsupported gene-peak pairs, scHPGT reduces the hypothesis space of possible alignments and encourages regulatory-consistent integration. Conceptually, the regulatory mask acts as a domain-specific grammar that restricts allowable relationships, analogous to the way structured linguistic priors can constrain representation learning in natural-language analysis (Kang *et al.* 2020).

A prior consistency objective further encourages the latent representation to respect regulatory constraints:


(5) $$L_{\mathrm{prior}}=\sum_{g=1}^{G}\sum_{p=1}^{P}M_{g,p}\cdot \text{dist}(q_{g},k_{p})$$

Here $q_{g}$ and $k_{p}$ denote the projected low-dimensional embeddings of gene *g* and peak *p*, respectively. The dissimilarity term dist(·,·) is computed between the corresponding latent RNA and ATAC feature representations and is weighted by $M_{g,p}$, so that high-confidence prior-supported pairs contribute more strongly to the regularization. Supplementary Methods report the exact latent-space dissimilarity, prior-normalization scheme, and mask-projection procedure. This separation keeps the main text focused on the model objective while preserving reproducibility.

The attention output is combined with the RNA gene-token representation through a residual connection, followed by a feed-forward network and layer normalization:


(6) $$H^{\mathrm{joint}}=\text{LayerNorm}(H^{\mathrm{gene}}+\text{FFN}(H^{\mathrm{cross}}))$$

The feature-level output $H^{\mathrm{joint}}$ of the prior-guided Transformer is thus obtained from the cross-modal attention representation $H^{\mathrm{cross}}$ through an FFN, a residual connection, and layer normalization. For downstream cell-level integration, let $h_{i}^{\mathrm{joint}}$ denote the cell-specific regulatory vector derived from $H^{\mathrm{joint}}$ for RNA cell *i*. This vector is concatenated with the modality-specific latent embedding $z_{i}^{\mathrm{rna}}$ and projected to form the final joint cell representation supplied to the GRL module:


(7) $$z_{i,\text{rna}}^{\mathrm{joint}}=\text{LayerNorm}\left(W_{f}\left[z_{i}^{\mathrm{rna}};h_{i}^{\mathrm{joint}}\right]+b_{f}\right)$$

Similarly, for ATAC cell *j*, the joint cell representation is:


(8) $$z_{j,\text{atac}}^{\mathrm{joint}}=\text{LayerNorm}\left(W_{f}\left[z_{j}^{\mathrm{atac}};h_{j}^{\mathrm{joint}}\right]+b_{f}\right)$$

where [·;·] denotes vector concatenation, and $W_{f}$ and $b_{f}$ are the learnable weight matrix and bias vector of the linear projection layer, respectively. The resulting joint cell representations are used as inputs to the domain-adversarial alignment module.

### 2.5 Domain-adversarial modality alignment

Because the data are unpaired, modality-specific signals can dominate the shared latent space. scHPGT therefore uses a domain discriminator $D_{\mathrm{dom}}$ together with a gradient reversal layer (GRL). The discriminator operates on the aggregated cell-level shared representations rather than directly on the gene-by-peak attention matrix. The discriminator is trained to distinguish RNA from ATAC embeddings, whereas the encoders are trained to confuse the discriminator. The adversarial loss is:


(9) $$\begin{array}{cc} L_{\mathrm{adv}}=-E_{z∼P_{\mathrm{rna}}}[\text{log}\;D_{\mathrm{dom}}(z_{\mathrm{rna}}^{\mathrm{joint}})] & \\ -E_{z∼P_{\mathrm{atac}}}[\text{log}(1-D_{\mathrm{dom}}(z_{\mathrm{atac}}^{\mathrm{joint}}))] & \end{array}$$

where $P_{\mathrm{rna}}$ and $P_{\mathrm{atac}}$ denote the distributions of latent representations for RNA and ATAC modalities, respectively.

During backpropagation, the GRL reverses the gradient before it reaches the encoders, encouraging modality-invariant representations (Ganin and Lempitsky 2015). In scHPGT, adversarial alignment operates within a representation already constrained by regulatory priors. Thus, the prior mask determines biologically plausible interactions, whereas adversarial learning reduces residual distributional discrepancy.

### 2.6 Optimization objective

The total objective combines reconstruction, prior consistency, and adversarial alignment:


(10) $$L_{\mathrm{total}}=\alpha L_{\mathrm{rec}}+\beta L_{\mathrm{prior}}+\gamma L_{\mathrm{adv}}$$

where hyperparameters α,β,γ balance information preservation, prior adherence and modality alignment. Excessively strong prior constraints can over-regularize the latent space, whereas overly strong adversarial alignment can suppress biologically meaningful variation. Parameter sensitivity analyses were therefore performed across representative datasets.

### 2.7 Extracting attention weights for regulatory validation

To interpret the learned cross-modal relationships, attention weights are extracted from the prior-guided Transformer after model training. For multi-head attention, attention matrices are averaged across heads and, when multiple Transformer layers are used, across the relevant cross-modal layers. The resulting gene-peak score matrix is mapped back from latent attention indices to the original gene and peak identifiers using the same feature dictionaries used to construct the prior mask.

For each gene, peaks are ranked by the corresponding attention-derived scores. Top-ranked gene-peak pairs are then evaluated against independent or held-out regulatory evidence, including external peak-gene links, cCRE overlap, motif enrichment, and attention-derived regulatory activity. For cell-type-specific analyses, attention scores are aggregated within each annotated cell type before ranking peaks. Detailed extraction, background matching, and statistical testing procedures are described in the Supplementary Methods.

## 3 Results

We evaluated scHPGT across four unpaired RNA-ATAC integration settings: PBMC3k, mouse spleen, CITE-seq/ASAP-seq PBMC, and PBMC10k. Baselines included gene-activity-based approaches (LIGER, SCALEX and uniPort) and peak-aware methods (GLUE and Cobolt). We assessed clustering agreement (ARI, NMI, and F1), modality mixing (BE), biological structure preservation (SI), label transfer, and regulatory interpretability.

### 3.1 scHPGT improves alignment while preserving cellular structure

We first benchmarked scHPGT on the unpaired PBMC3k RNA-ATAC dataset (Fig. 2A–C). In the integrated embeddings, scHPGT and GLUE achieved the strongest overall alignment, but their biological structure differed (Fig. 2A). GLUE and uniPort mixed modalities effectively, whereas scHPGT produced more compact and well-defined cell-type clusters. Cobolt and SCALEX showed less precise alignment, and LIGER struggled to capture a coherent shared representation.

**Figure 2:**
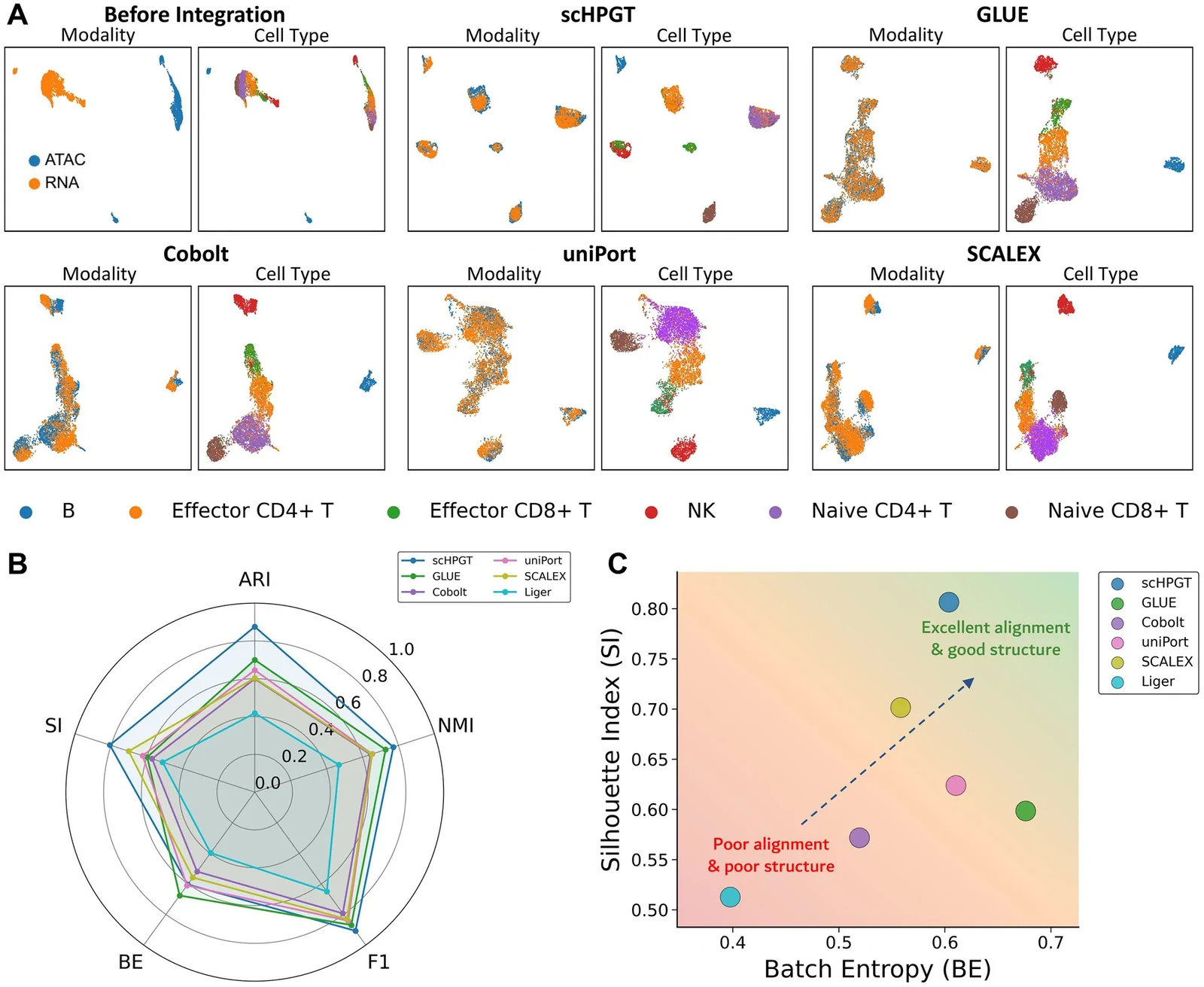
scHPGT balances modality mixing and biological structure preservation. (A) UMAP embeddings of PBMC3k before and after integration, colored by modality and cell type. (B) Multi-metric radar summary of ARI, NMI, F1, BE and SI across methods. (C) Trade-off between batch entropy (BE) and silhouette index (SI), showing that scHPGT achieves strong modality mixing while preserving biological structure.

The quantitative comparison supported this visual assessment (Fig. 2B). scHPGT achieved the highest ARI, NMI, F1, and SI on PBMC3k, while trailing GLUE only modestly in BE. This trade-off is important because high BE alone can indicate strong modality mixing without guaranteeing preservation of cellular identity. The BE-SI analysis placed scHPGT in the most favorable region, combining effective modality alignment with superior biological separation (Fig. 2C).

The mouse spleen benchmark supported the same conclusion (Fig. S1A–C). Although GLUE and uniPort showed comparable mixing, their embeddings were more diffuse for several cell populations, including B transitional and granulocyte populations (Fig. S1A). scHPGT produced a more structured embedding and achieved the best overall performance across ARI, NMI, F1, BE, and SI (Fig. S1B and C). These results suggest that regulatory-constrained attention improves integration without sacrificing fine-grained cell-state structure. We additionally evaluated the capability of scHPGT to integrate paired datasets by collecting paired RNA and ATAC data across different species and scales, as detailed in Supplementary Methods, line 131.

### 3.2 Regulatory priors reshape attention into biologically consistent interactions

To understand why scHPGT improves integration performance, we examined the behavior of the attention mechanism (Fig. 3A–C). Before prior masking, the raw attention matrix contained many dense and weakly structured gene-peak associations (Fig. 3A, left). After prior-mask filtering, attention became sparse and concentrated on biologically plausible regulatory links (Fig. 3A, right). To clearly illustrate how the prior-guided attention mechanism operates, we specifically filtered for genes and peaks with established regulatory relationships from the biological prior and ordered them by genomic coordinates. This restructuring indicates that the prior does not merely provide a static feature graph but actively constrains cross-modal information flow.

**Figure 3:**
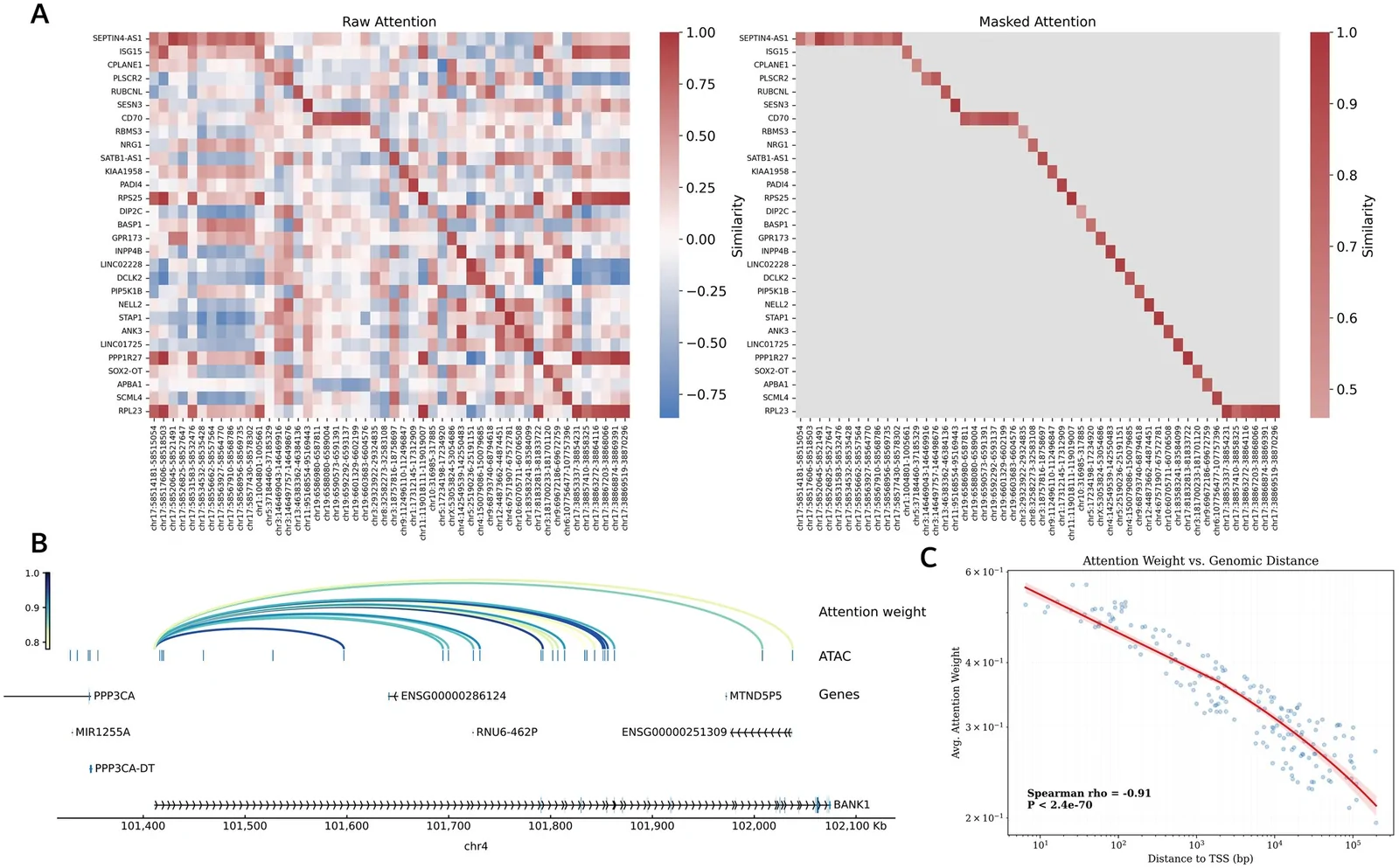
Regulatory priors guide biologically consistent attention. (A) Gene-peak attention patterns before and after prior-mask processing. (B) Genome-track visualization of masked attention at the *BANK1* locus. (C) Relationship between average attention weight and distance to the transcription start site (TSS), showing distance-dependent attenuation of attention strength.

Genome-track visualization at the BANK1 locus further illustrated this mechanism (Fig. 3B). Peaks prioritized by scHPGT attention overlapped accessible regions and connected BANK1 to local chromatin features in a manner consistent with cis-regulatory structure. Across genes, average attention weights decreased as distance to the transcription start site increased (Fig. 3C), consistent with enrichment of promoter-proximal regulatory influence. These analyses establish the mechanistic role of the prior-guided Transformer: it filters noisy gene-peak associations and concentrates cross-modal attention on regulatory-consistent interactions.

### 3.3 scHPGT attention captures biologically meaningful regulatory signals

We next asked whether regulatory links prioritized by scHPGT correspond to biological evidence beyond the model architecture (Fig. 4A–F). For representative marker genes, top attention peaks localized to accessible gene-associated genomic regions (Figs. 4A and S3). These locus-level examples provide interpretable evidence that high-attention peaks correspond to plausible regulatory elements rather than arbitrary local features.

**Figure 4:**
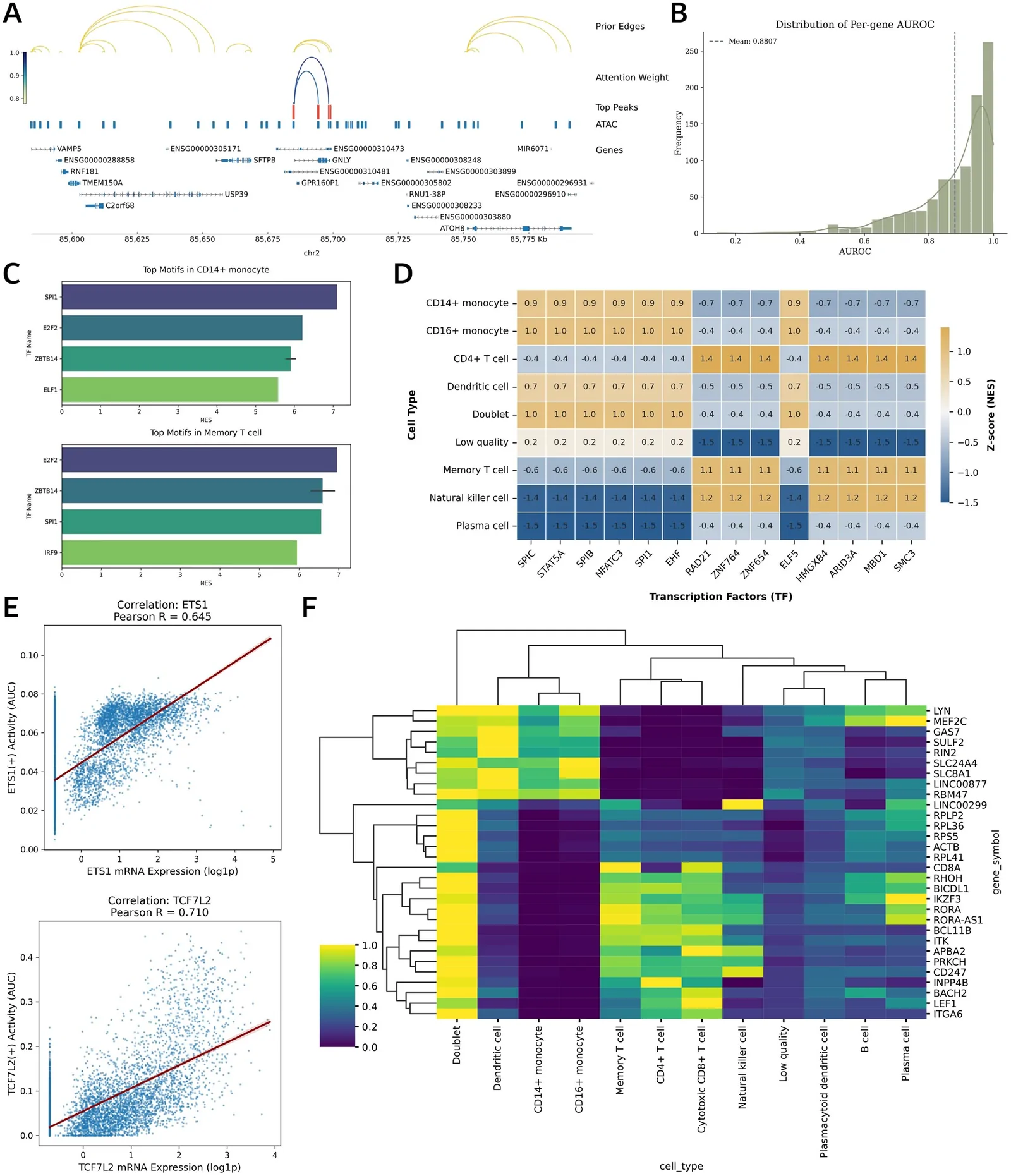
scHPGT attention captures regulatory relationships supported by biological evidence. (A) Genome-track example of high-attention peaks for a representative marker gene, showing prior edges, attention weights, selected top peaks, accessibility signal, and gene annotation. (B) AUROC distribution for gene-associated top peaks using independently inferred gene-peak links as reference. (C) Top motif enrichment inferred from high-attention peaks in representative cell types. (D) Differential transcription factor enrichment across cell types after cross-cell-type normalization. (E) Correlation between attention-derived regulatory activity and mRNA expression for marker transcription factors. (F) Cell-type-specific regulatory activity programs inferred from scHPGT attention.

Using independently inferred gene-peak regulatory relationships as a reference, top attention links achieved high recovery performance (mean AUROC = 0.8807; Fig. 4B). Enrichment analyses further showed that attention-derived links were markedly enriched over randomized gene-peak relationships (Fig. S4A and B). Functional annotation of high-attention peaks using ENCODE SCREEN candidate cis-regulatory element (cCRE) annotations showed strong enrichment across regulatory categories, including promoter-like and distal enhancer-like elements (Fig. S4C and D) (Moore *et al.* 2026).

Motif analyses linked high-attention peaks to transcription factor programs. The top enriched motifs included immune regulators and broadly active factors such as *SPI1*, *E2F2*, *ZBTB14* and *IRF9* (Figs. 4C and S5). Cross-cell-type normalization, together with established single-cell regulatory-network analysis concepts such as SCENIC, revealed more specific regulatory modules (Aibar *et al.* 2017): myeloid populations showed enrichment of *SPIC*, *SPIB*, *STAT5A*, and *NFATC3*, whereas lymphoid lineages showed enrichment of cohesin-associated and zinc-finger factors including *RAD21*, *SMC3*, *ZNF764*, and *ZNF654* (Fig. 4D).

Finally, attention-derived regulatory activity correlated with matched mRNA expression for lineage-associated transcription factors (Fig. 4E). *TCF7L2* and *ETS1* showed strong positive correlations between regulatory activity and expression, and additional marker genes showed similar trends (Fig. S6). Cell-type-specific regulatory activity profiles recovered canonical programs, including *EBF1* activity in B cells and plasma cells, LYN-associated activity in monocytes, and coordinated lymphoid activity involving *CD8A*, *CD247*, *LEF1*, and *IKZF3* (Fig. 4F). Together, these analyses connect integration to regulatory interpretation and support scHPGT as a biologically meaningful representation model, complementing transcriptome-based regulatory-network inference frameworks (Aibar *et al.* 2017).

### 3.4 scHPGT avoids spurious alignment under missing cell populations

We next evaluated robustness to partial-overlap cell-type compositions using PBMC10k (Figs. 5A–C and S7A–C). In the Unbalance-ATAC setting, selected cell types were removed from ATAC while retained in RNA; in the Unbalance-RNA setting, the same populations were removed from RNA while retained in ATAC. These designs test whether a model can align shared cell states without forcing modality-specific populations into incorrect matches.

**Figure 5:**
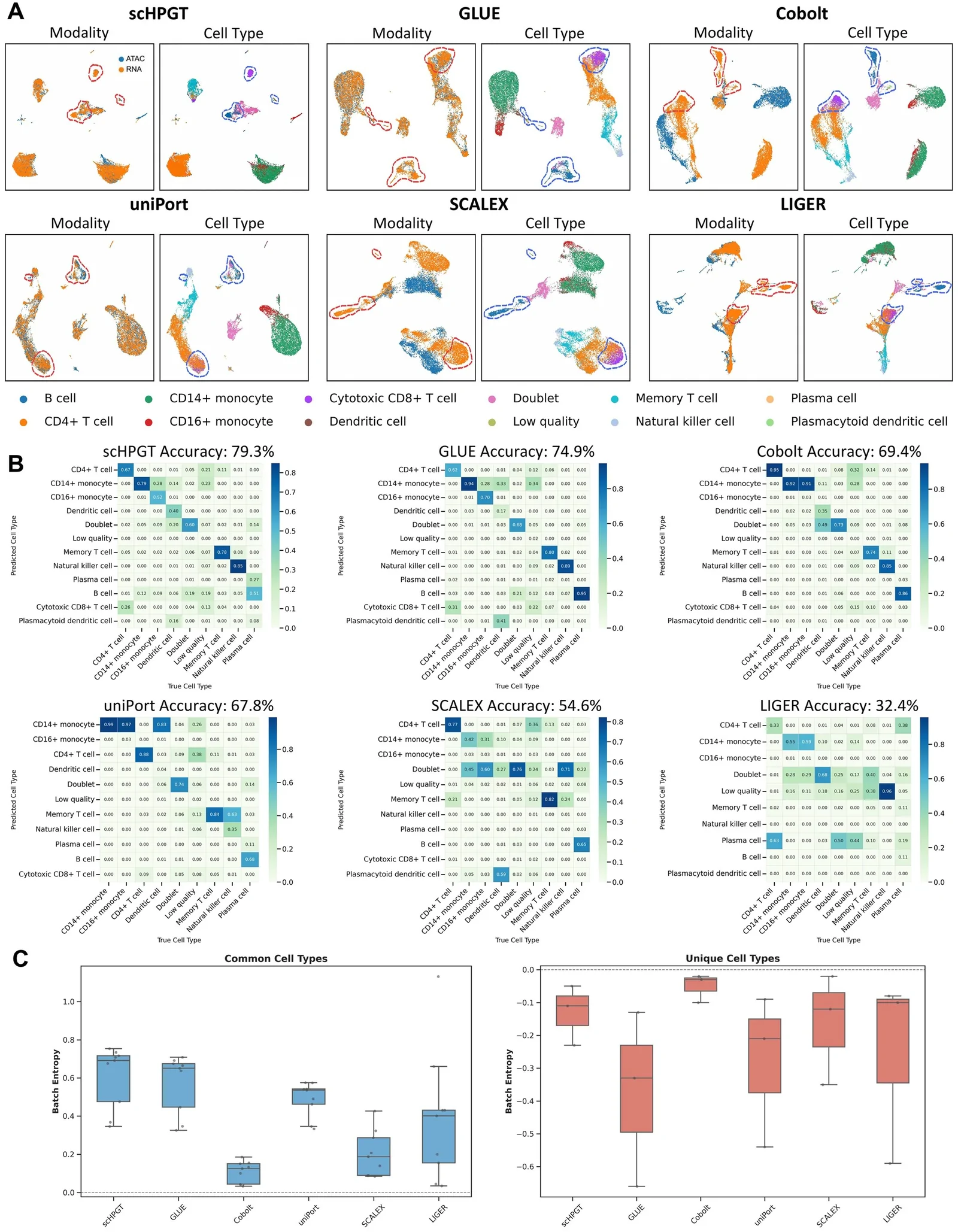
scHPGT avoids spurious alignment under missing cell populations. (A) UMAP embeddings under the Unbalance-ATAC setting, colored by modality and cell type; red and blue outlines mark populations with missing cross-modal counterparts. (B) Label-transfer confusion matrices across methods, with average transfer accuracy indicated for each method. (C) Local batch entropy for common and unique cell groups. Higher values in common groups indicate better alignment of shared states, whereas values closer to zero in unique groups indicate better preservation of modality-specific populations.

Under Unbalance-ATAC, scHPGT preserved distinct clusters for cell types with missing counterparts while maintaining alignment among shared cell populations (Fig. 5A). B cells remained close to but distinct from doublets, and cytotoxic CD8+ T cells formed distinguishable groups. GLUE showed partial co-localization of B cells and doublets, and other methods showed weaker alignment or more diffuse cell-type separation.

Label transfer results further supported the robustness of scHPGT (Fig. 5B). scHPGT achieved the highest average accuracy (79.3%) and more reliable discrimination of closely related or underrepresented populations. In contrast, uniPort and Cobolt more frequently confused CD14+ and CD16+ monocytes, and SCALEX and LIGER had substantially weaker transfer performance. In the reciprocal Unbalance-RNA setting, scHPGT again achieved the highest average label-transfer accuracy (70.7%) and accurately transferred CD16+ monocytes, whereas other methods frequently misclassified them as CD14+ monocytes (Fig. S7B).

We separated common cell types from unique cell types and evaluated local batch entropy with a mismatch penalty for unique populations. In the common group, higher values indicate stronger alignment of shared cell states; in the unique group, values closer to zero indicate better preservation of modality-specific populations. scHPGT achieved strong alignment among common groups while avoiding spurious mixing of unique groups in both Unbalance-ATAC (Fig. 5C) and Unbalance-RNA (Fig. S7C). scHPGT consistently outperformed competing methods across ARI, NMI, F1, and SI, with a minor exception on the Unbalanced-RNA dataset where its BE was marginally lower than that of GLUE (ΔBE=0.73, Fig. S8). These results demonstrate selective alignment: scHPGT aligns shared biological states while preserving cell populations that lack cross-modal counterparts.

### 3.5 scHPGT preserves condition-specific differences during cross-condition integration

When cells are in different conditions, their underlying transcriptomic and epigenetic profiles undergo substantial shifts. Without alignment, cells from these different conditions completely segregate in the latent space due to overwhelming background expression shifts and platform-specific batch effects (e.g. stimulated T cells and control T cells reside at opposite ends of the space). The objective of alignment is to construct a shared biological backbone (cell-type identity) that links homologous populations across conditions.

We further evaluated scHPGT in a cross-condition setting using stimulated CITE-seq RNA data and unstimulated ASAP-seq ATAC data (Figs. S9A–C and S10A, B). The RNA condition used anti-CD3/CD28 stimulation in the presence of IL-2, a standard T-cell activation setting (Martkamchan *et al.* 2016), whereas the ATAC data came from control ASAP-seq profiles generated in a multimodal profiling framework (Mimitou *et al.* 2021). This experiment introduces a biological condition shift, particularly among T-cell populations, and therefore tests whether integration can preserve condition-specific variation rather than forcing indiscriminate mixing.

scHPGT aligned major immune populations while maintaining separation among T-cell subtypes (Fig. S9A). CD4+ and CD8+ T cells formed well-resolved groups, and cells from different modalities were brought into a shared space without complete collapse of condition-specific differences. GLUE and uniPort achieved strong modality mixing but showed less distinct T-cell boundaries, whereas SCALEX, Cobolt, and LIGER showed weaker alignment or poorer biological separation.

Label transfer showed that scHPGT and GLUE achieved the highest accuracies (approximately 89.3% and 87.6%, respectively), outperforming uniPort, Cobolt, SCALEX, and LIGER (Fig. S9B). Silhouette profiles confirmed that scHPGT produced more cohesive and well-separated biological clusters (Fig. S9C). Although GLUE achieved the highest BE (Fig. S10B), this likely reflects stronger mixing of stimulated and control states rather than a clear biological advantage. scHPGT therefore, better balances integration with preservation of condition-specific regulatory divergence.

### 3.6 scHPGT preserves fine-grained structures and rare cell populations

To comprehensively evaluate scHPGT beyond macro-level discrete clustering metrics, we assessed its capability to preserve fine-grained biological structures, maintain continuous manifold alignment, and protect rare cell populations across both balanced and imbalanced multimodal datasets.

We identified rare cell types across all benchmark datasets (defined as populations comprising less than 10% of the total cell count) and evaluated their alignment quality using KNN label transfer accuracy (Fig. 6). In standard balanced datasets (PBMC3k, Mouse Spleen, and CITE/ASAP-seq), scHPGT demonstrates consistently superior protection for low-abundance populations. On PBMC3k, scHPGT achieves label transfer accuracy scores of 0.98 for B cells and 0.83 for Effector ${\text{CD}8}^{+}$ T cells, outperforming GLUE. For Dendritic cells in the Mouse Spleen dataset, while LIGER suffers severe misalignment (0.02), scHPGT maintains a high accuracy of 0.98. In the highly imbalanced Unbalanced-ATAC dataset, methods relying purely on global distribution matching exhibit catastrophic performance collapses on rare populations; specifically, SCALEX and LIGER drop to an accuracy of 0.00 on Dendritic cells, while GLUE completely misaligns Plasma cells, also resulting in an accuracy of 0.00. Remarkably, scHPGT achieves optimal performance across almost all metrics without experiencing any zero-accuracy failures, thereby attaining superior global alignment. These results indicate that the prior masking constraint architecture in scHPGT effectively preserves native intra-modality biological structures.

**Figure 6:**
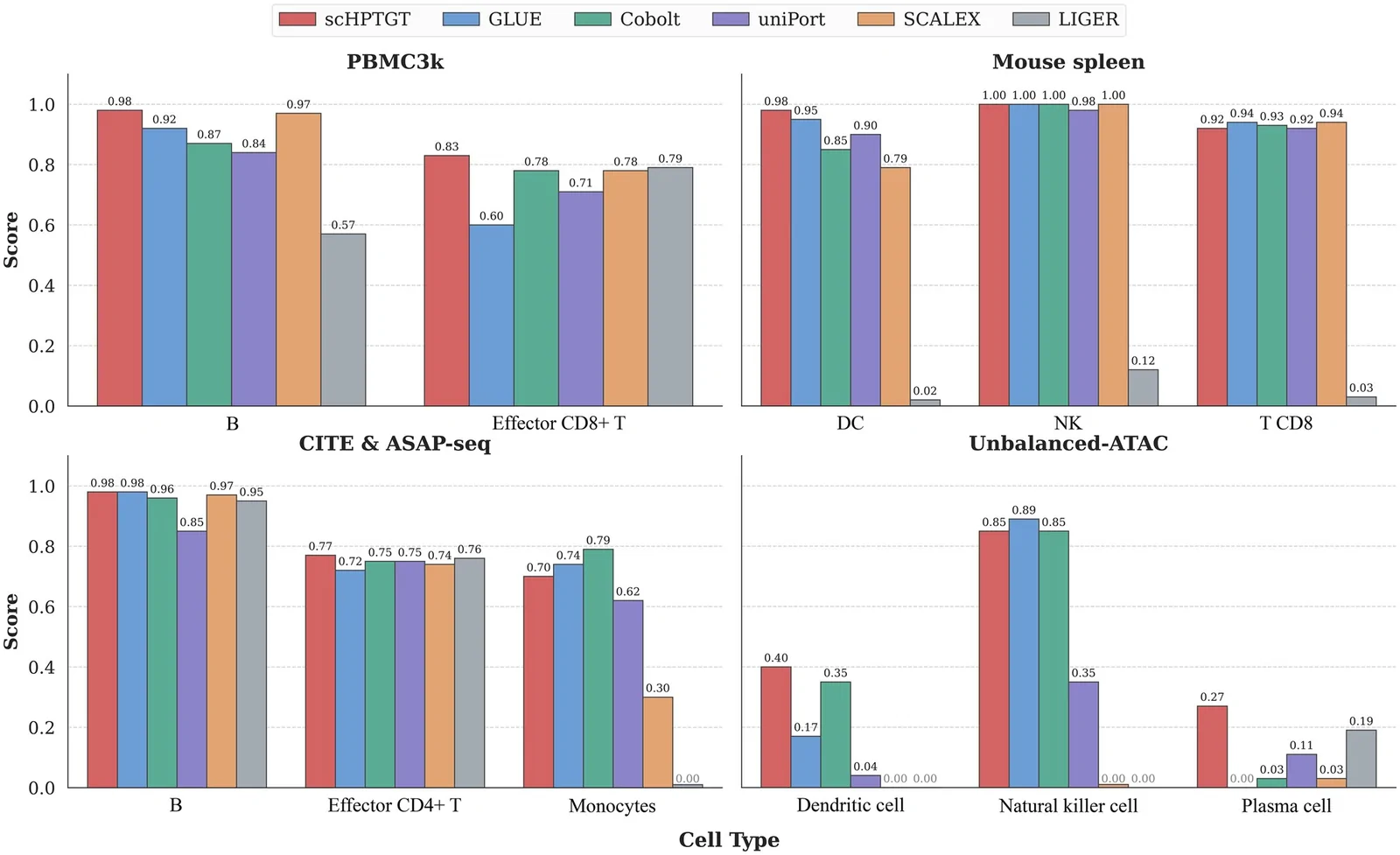
Evaluation of label transfer accuracy for rare cell types across datasets.

We further leveraged the standard scib (Luecken *et al.* 2022) package to quantify continuous biological preservation and batch mixing via cLISI and iLISI metrics (Fig. 7A), respectively. Following the standard scib pipeline, raw LISI scores were normalized to [0,1] using min-max scaling, where values closer to 1.0 indicate superior performance across all reported metrics. For highly imbalanced datasets (Unbalanced-RNA and Unbalanced-ATAC), we additionally computed the Isolated Label Silhouette Width (isolated_labels_asw) to assess the model’s capability to preserve and segregate isolated cell labels present in only one modality (Fig. 7B).

scHPGT obtains high and consistent cLISI scores (0.990–0.997) across all benchmark datasets, indicating that it effectively preserves local cell-type structures and prevents over-smoothing during cross-modal integration. Regarding multimodal alignment (iLISI), scHPGT demonstrates strong integration performance, particularly yielding higher iLISI scores on Mouse Spleen (0.998), Unbalanced-RNA (0.928), and Unbalanced-ATAC (0.939) compared to baseline methods. While GLUE achieves a higher iLISI on PBMC3k (0.789 vs. 0.709), scHPGT maintains a more balanced trade-off between biological structure preservation and batch alignment. Furthermore, on the unbalanced datasets, scHPGT achieves ASW scores of 0.880 on Unbalanced-RNA and 0.916 on Unbalanced-ATAC (Fig. 7B), markedly outperforming baseline methods (e.g. GLUE at 0.633 and 0.675). This confirms that the prior masking constraint in scHPGT assists in maintaining representation separation for low-abundance and modality-specific cell types during domain-adversarial training.

**Figure 7:**
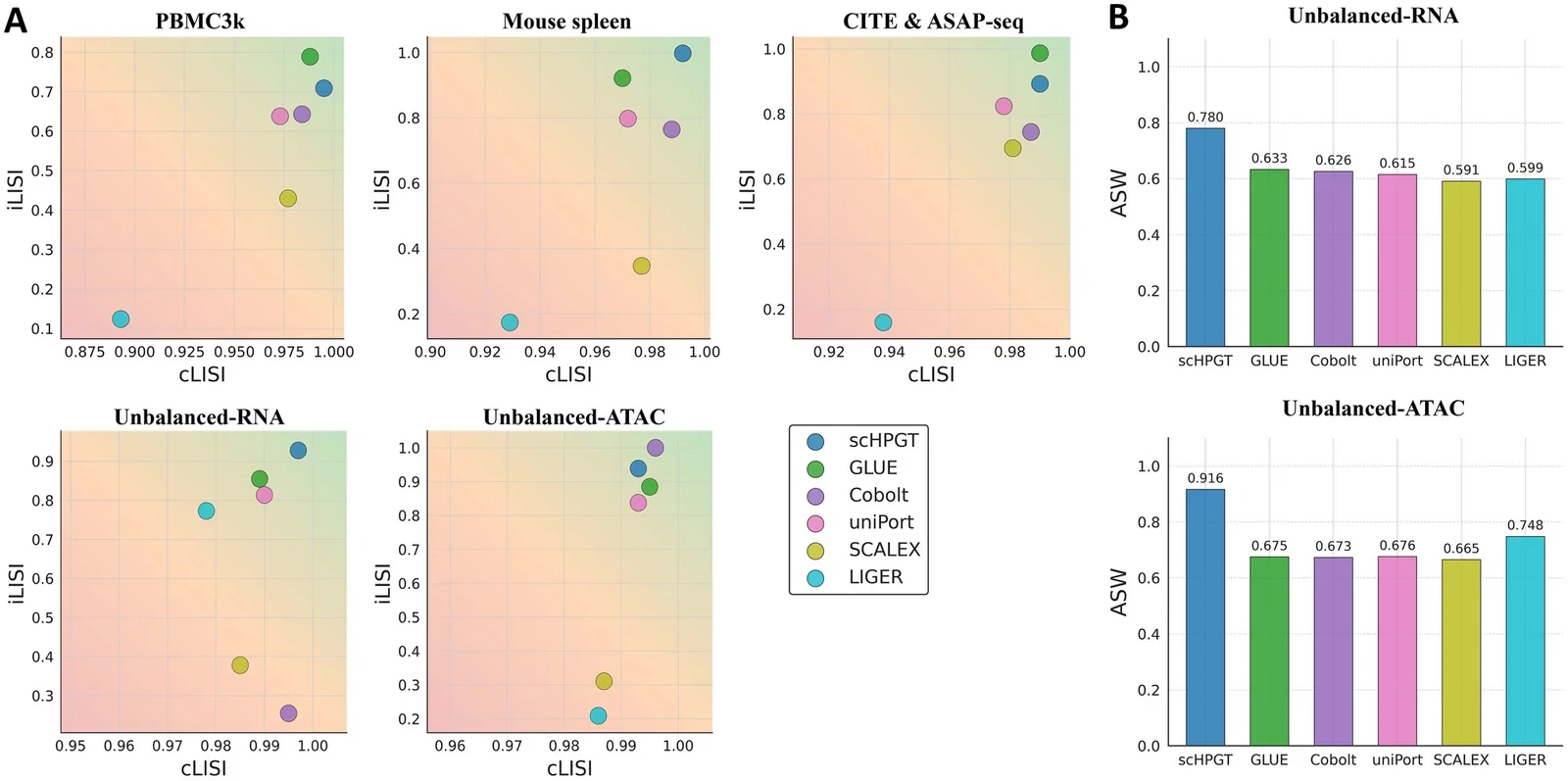
Benchmark of fine-grained local structure and batch alignment performance across diverse datasets. (A) cLISI vs. iLISI scatter plots for five datasets. (B) Isolated label ASW scores on Unbalanced-RNA and Unbalanced-ATAC datasets.

### 3.7 Prior guidance and adversarial alignment play complementary roles

To disentangle the roles of the principal components of scHPGT, we performed prior perturbation and adversarial ablation experiments. Perturbing or removing the prior matrix reduced biological structure preservation and regulatory interpretability (Fig. 8A–C). The full prior achieved the best overall performance across integration metrics (Fig. 8A). Dropping prior edges, adding random edges or shuffling the prior progressively degraded performance. The shuffled-prior setting performed worse than the no-prior baseline, indicating that incorrect priors can actively misguide alignment. We performed additional ablation experiments targeting long-range edges and the edge scoring method, with further details provided in Supplementary Methods (line 109).

**Figure 8:**
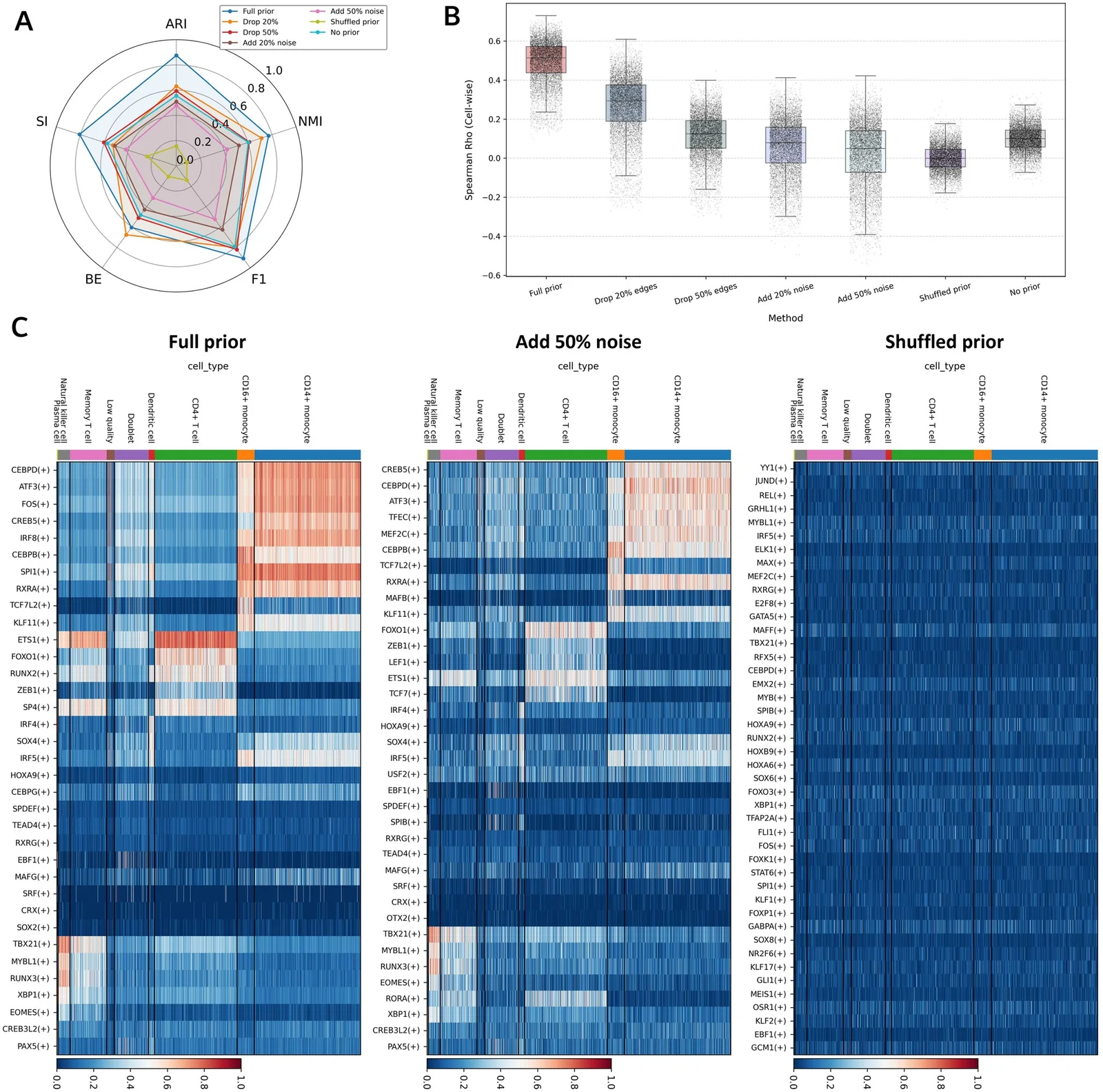
Biologically meaningful prior structure is required for regulatory-consistent integration. (A) Integration metrics across prior-processing strategies, including full prior, edge dropout, noise addition, shuffled prior, and no-prior settings. (B) Correlation between attention-derived regulatory activity and gene expression under prior perturbation. (C) Motif-enrichment patterns under full, noisy, and shuffled priors, showing loss of structured regulatory programs when the prior is corrupted.

Regulatory activity and motif analyses confirmed this interpretation. Full-prior attention produced the strongest correlation between regulatory activity and matched mRNA expression (Fig. 8B) and recovered clear cell-type-specific motif programs (Fig. 8C). No-prior and shuffled-prior settings produced disorganized or near-zero enrichment signals (Fig. S11). These results show that the prior mask functions as a biologically meaningful constraint rather than a generic regularizer.

Removing the GRL produced a different failure mode (Fig. 9A–C). Without adversarial alignment, RNA and ATAC embeddings remained separated by modality across PBMC3k, mouse spleen, and CITE-seq/ASAP-seq datasets (Fig. 9A–C, left and middle panels). Quantitative radar summaries showed reduced modality mixing and lower label-transfer performance (Fig. 9A–C, right panels). Thus, prior guidance controls which cross-modal interactions are biologically plausible, whereas adversarial alignment controls how modalities are mixed in latent space. Together, these components enable biologically faithful integration.

**Figure 9:**
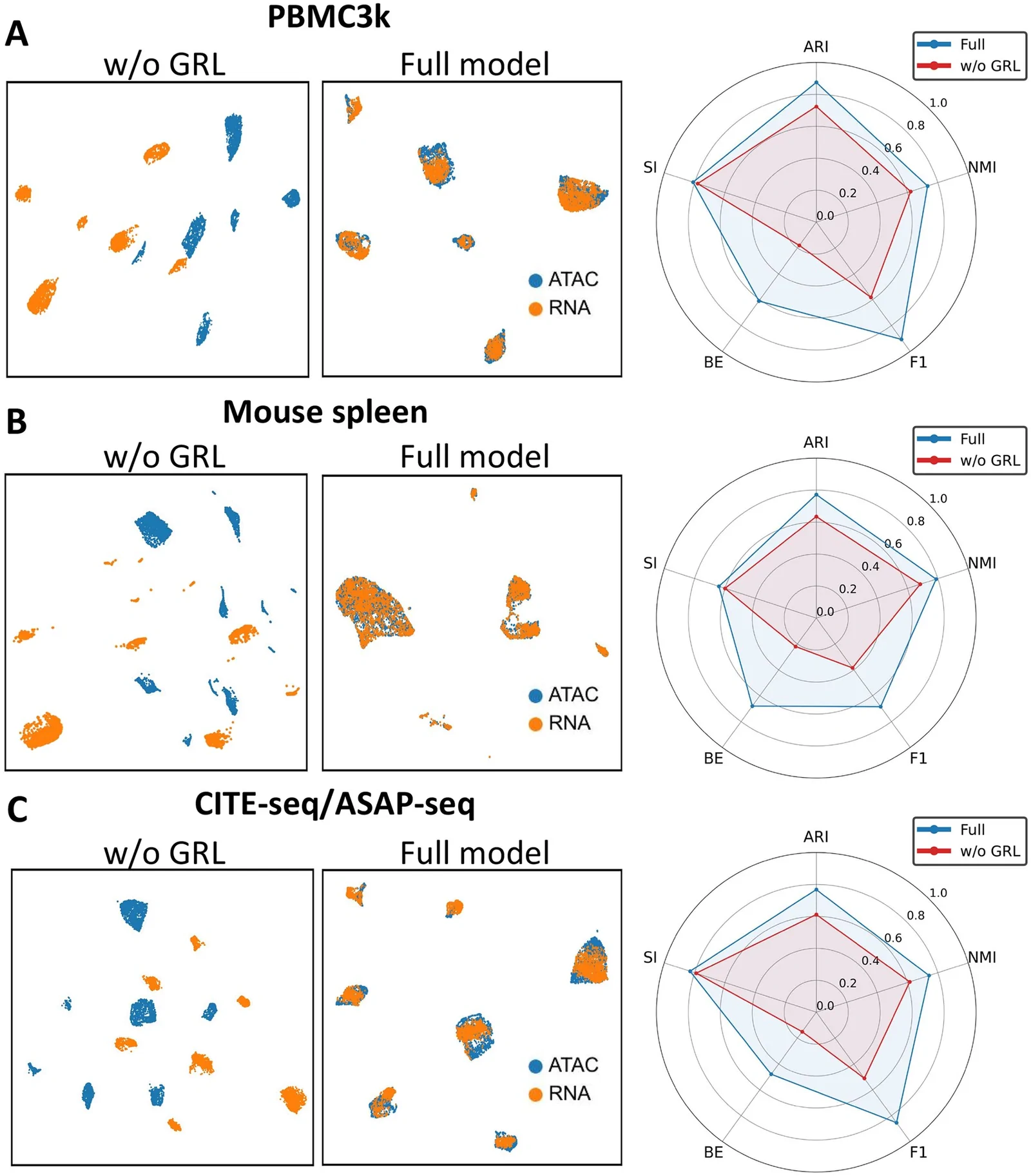
Domain-adversarial alignment is required for modality mixing. A-C UMAP embeddings and radar summaries for PBMC3k (A), mouse spleen (B) and CITE-seq/ASAP-seq PBMC (C). Removing the GRL separates RNA and ATAC modalities and reduces integration performance, whereas the full model improves modality mixing while preserving biological structure.

### 3.8 Parameter sensitivity analysis

We assessed the sensitivity of scHPGT to the prior weight β, adversarial weight γ, and latent dimension *d* across PBMC3k, CITE-seq/ASAP-seq PBMC, and mouse spleen datasets (Supplementary Figs. S13–S16 for UMAP clustering results and Fig. S17 for quantitative integration metrics).

Insufficient prior guidance (β=1) resulted in fragmented clustering and substantial performance degradation, with ARI falling below 0.64 for both PBMC3k and CITE-seq/ASAP-seq PBMC. Conversely, overly stringent regularization (β=30) over-constrained the latent space and attenuated biological heterogeneity, leading to decreases in ARI, NMI, F1, and SI across all three datasets. Across the tested configurations, β=5 consistently provided a stable balance between data-driven representation learning and prior-guided regularization.

The adversarial weight γ exhibited a similar trade-off in modality alignment. Insufficient adversarial pressure (γ=0.3) compromised modality mixing, with BE decreasing below 0.35 in the mouse spleen dataset. In contrast, excessive adversarial alignment (γ=5.0) suppressed biological structure, causing ARI to fall below 0.50 in PBMC3k. Overall, γ=0.7 provided the most robust integration performance among the tested values, balancing cross-modality alignment with biological structure preservation.

Latent dimensions below 64 (d=16 or 32) were generally insufficient to capture fine-grained cellular heterogeneity; at d=16, all evaluated metrics for PBMC3k were below 0.50. The effect of latent dimensionality was dataset-dependent. Performance on the mouse spleen dataset largely saturated at d=64, and the CITE-seq/ASAP-seq PBMC dataset achieved its highest ARI and F1 at this intermediate dimension. PBMC3k benefited from greater representational capacity, achieving its highest clustering performance at d=128 (ARI = 0.875). At dimensions of 256 or greater, performance gains were marginal while optimization became more difficult. We therefore selected d=128 as the default latent dimension to balance representational capacity and computational efficiency across multi-omics integration tasks.

## 4 Discussion

scHPGT demonstrates that unpaired single-cell multi-omics integration benefits from explicitly coupling distributional alignment with regulatory structure. Existing approaches often focus on matching latent distributions or harmonizing feature spaces. scHPGT instead formulates integration as biologically constrained alignment, in which peak-gene priors restrict cross-modal attention and adversarial learning reduces residual modality-specific variation.

The results support three conclusions. First, regulatory-prior-guided attention improves integration quality by reducing spurious gene-peak interactions and preserving cell-type structure. Second, the learned attention is biologically interpretable: it recovers gene-peak links, enriches for functional regulatory elements and motifs, and produces regulatory activity consistent with transcriptional programs. Third, scHPGT is robust in difficult settings, including unbalanced cell-type composition and cross-condition integration, where indiscriminate mixing can obscure meaningful biological differences. In addition, scHPGT generalizes effectively to paired multimodal datasets across diverse scales and species, achieving seamless cross-modal alignment while accurately discriminating fine-grained cell types.

A key conceptual insight is the separation of what to align from how to align. The prior-guided Transformer defines biologically plausible cross-modal interactions, whereas the domain-adversarial module aligns modalities within the constrained representation space. This division helps prevent two common failure modes: biologically implausible over-alignment and residual modality-driven separation.

Several limitations remain. scHPGT depends on the quality and coverage of the peak-gene prior graph; incomplete or inaccurate priors can reduce interpretability and may actively mislead alignment when strongly corrupted. Although the model remained robust to moderate prior perturbation, future work should explore adaptive prior refinement and uncertainty-aware prior weighting. Initial insights from our prior graph ablation—where replacing binary adjacency with correlation-based continuous scoring yielded performance gains—indicate that developing a principled, continuous edge-scoring strategy represents a crucial direction for future optimization. The computational cost of attention over high-dimensional peak features also motivates sparse or hierarchical attention for very large atlases. Finally, additional orthogonal validation using experimentally verified enhancer-gene maps, promoter-capture Hi-C, eQTLs or perturbation datasets would further strengthen biological interpretation.

More broadly, scHPGT suggests a path toward interpretable multi-omics representation learning. By embedding regulatory structure directly into cross-modal attention, the framework can complement emerging pretrained single-cell models and can be extended beyond RNA-ATAC integration to protein, spatial, or perturbational modalities (Cui *et al.* 2024). Such extensions may help bridge single-cell integration, regulatory network inference, and mechanistic interpretation of cell-state variation.

## 5 Data preprocessing

We evaluated scHPGT on PBMC3k, mouse spleen, CITE-seq/ASAP-seq PBMC, and PBMC10k datasets. For gene-centric integration methods, scATAC-seq peak counts were converted into gene activity features using Signac (Stuart *et al.* 2021). For peak-based methods, peak count matrices were processed following the GLUE workflow (Cao and Gao 2022), including quality control, TF-IDF normalization, and latent semantic indexing.

- *PBMC3k*: The PBMC3k dataset was obtained from UniPort. The processed and annotated scRNA-seq data contain 3846 cells across 17 256 genes, while the scATAC-seq data were transformed into a gene activity matrix by Signac, containing 3254 cells with 24 138 genes.
- *Mouse spleen*: The mouse spleen scATAC-seq dataset was retrieved from ArrayExpress. Raw reads across all cells were pooled to enable robust peak calling using MACS2 (Grytten *et al*. 2019), thereby identifying regions of open chromatin. The resulting cell-by-bin accessibility matrix was binarized and subjected to latent semantic indexing (LSI) for dimensionality reduction (Cusanovich *et al*. 2015). Specifically, the binarized data underwent TF–IDF (term frequency–inverse document frequency) transformation, using term frequency combined with smoothed inverse document frequency to normalize for sequencing depth and feature scarcity. Subsequently, singular value decomposition (SVD) was applied to the TF–IDF-transformed matrix to extract the most informative low-dimensional components for downstream analysis.
- *CITE-seq and ASAP-seq PBMC data*: This dataset was downloaded from the GEO database (accession: GSE156478) (Mimitou *et al*. 2021). It includes ASAP-seq fragment files and antibody-derived tag (ADT) matrices from the control condition (scATAC-seq), as well as the raw ADT matrix from CITE-seq under the stimulation condition (scRNA-seq). All data were preprocessed using Signac before integration.
- *PBMC10k*: The PBMC10k dataset was downloaded from the 10× Genomics website. For chromatin accessibility analysis, we aligned reads from both scATAC-seq and the unsorted Multiome data to a peak set identified from Multiome Chromium X data. During downstream clustering, clusters with high mitochondrial gene expression were identified and annotated as low-quality cell clusters. In addition, if the majority of cells within a cluster were detected as doublets by DoubletFinder, the cluster was annotated as a doublet cluster.

## 6 Baselines and parameter settings

- *uniPort*: Integrates adversarial learning with variational autoencoders (VAEs) to learn modality-invariant features in a shared latent space, enabling batch correction and preservation of biological variability without requiring paired samples. We set $\lambda_{s}=1.0$ and used UMAP visualization with parameters n_neighbors=30, use_rep=latent, and min_dist=0.1.
- *GLUE*: Integrates scRNA-seq and scATAC-seq by leveraging a peak–gene guidance graph to link features across modalities, and learns a shared cell embedding through graph-linked representation learning. We followed the official GLUE workflow for preprocessing and model training, and used the default parameter settings for integration and downstream embedding.
- *Cobolt*: Uses a multimodal variational autoencoder (VAE) to model scRNA-seq and scATAC-seq jointly and infer a shared latent representation for co-embedding and clustering. We formatted the input data as an MTX matrix together with a TSV feature file, as required by Cobolt. During feature filtering, we used upper_quantile=0.99 and lower_quantile=0.7. For model training, we set the learning rate to lr=0.007.
- *LIGER*: Employs integrative non-negative matrix factorization (iNMF) (Brunet *et al*. 2004) to identify shared and dataset-specific factors, enabling joint clustering across modalities after gene-centric feature harmonization (e.g. gene activity transformation for ATAC). We ran LIGER with default parameters and used the shared factor matrix for downstream visualization.
- *SCALEX*: Applies a decoupled learning strategy to separate biological signals from batch effects by embedding multi-omics data into a common latent space, enabling biologically faithful integration while reducing technical noise. We used the default parameter settings.

## Author contributions

Zhenglong Cheng (Data curation [equal], Methodology [equal], Validation [equal], Visualization [equal], Writing—original draft [equal]), Jiao Zhang (Investigation [equal], Project administration [equal], Validation [equal], Writing—review & editing [equal]), and Shixiong Zhang (Funding acquisition [lead], Investigation [lead], Project administration [Lead], Validation [lead], Writing—original draft [equal], Writing—review & editing [equal])

## Supplementary Material

btag696_Supplementary_Data

## Data Availability

PBMC3k was obtained from the UniPort resource. Mouse spleen data were retrieved from ArrayExpress (E-MTAB-6714) and are also available through the MultiMAP (Jain *et al.* 2021) dataset collection. CITE-seq/ASAP-seq PBMC data were downloaded from GEO accession GSE156478. PBMC10k data are available from the 10x Genomics website. The source code for scHPGT is available at https://github.com/zlCreator/scHPGT. The final release includes preprocessing scripts, random seeds, trained-model configuration files, and versioned package information to support reproducibility.
