# Supplementary material for: Regulatory-prior-guided attention preserves biological structure during unpaired single-cell RNA–ATAC integration
Source: Bioinformatics. 2026 Sep 18;42(10):btag696. doi: 10.1093/bioinformatics/btag696 (PMC13637587; doi:10.1093/bioinformatics/btag696)
Supplement: Supplementary file 1 [file btag696_supplementary_data.pdf]

Supplementary for scHPGT

Supplementary Methods

1. Data filtering and feature preprocessing

Low-quality scATAC-seq cells were removed if they had fewer than 1,800 reads or more than 100,000 reads. Low-quality scRNA-seq cells were removed if they had fewer than 1,000 unique molecular identifiers (UMIs) or more than 25,000 UMIs. To reduce cross-omics alignment bias caused by poor chromatin profiles, cells with nucleosome signal scores greater than 2 or transcription start site (TSS) enrichment scores less than or equal to 1 were excluded.

For RNA profiles and ATAC-derived gene activity matrices, counts were normalized and log transformed before selecting the top 2,000 highly variable genes and scaling the data. For peak-based ATAC analyses, peak count matrices were processed using TF-IDF normalization and latent semantic indexing following the GLUE-style workflow used in the main comparisons.

2. Peak-gene prior graph construction

The regulatory prior used by scHPGT is represented as a sparse gene-by-peak matrix M in $R^{G\times P}$, where G is the number of genes and P is the number of ATAC peaks retained after preprocessing. Each non-zero entry $M_{g,p}$ indicates that peak p is considered a plausible regulatory element for gene g.

Promoter-proximal links were assigned using gene annotations from the corresponding genome build. A peak was linked to a gene if the peak overlapped the gene body or promoter-proximal region around the gene transcription start site (TSS). We linked peaks to genes when the peak summit overlapped the gene body or was located within ±2 kb of the annotated TSS. Gene annotations were based on GENCODE v49 for human and GENCODE vM25 for mouse datasets. Coordinates were harmonized to hg38 (human) or mm10 (mouse) before constructing the prior matrix.

Distal links were defined using a distance-attenuation weight function, retaining peak–gene pairs within 150 kb with regulatory scores greater than 0.05. If no distal prior was available for a dataset, only promoter-proximal links were used (Table S1). The source of each link type is retained as metadata so that promoter-proximal and distal links can be evaluated separately.

Table S1: Peak-gene prior-link classes used by scHPGT

Prior-link class	Definition	Metadata recorded
Promoter-proximal	Peak overlaps the gene body or promoter/TSS window of a gene	Gene ID, TSS, peak coordinates, genome build, window size
Distal regulatory	Peak linked to a gene by long-range GLUE-style windowing or external regulatory annotation	Source method/database, distance-based weight or score, FDR or confidence value
Background/non-link	Gene-peak pair not supported by the prior	Retained as zero entry in the sparse prior matrix

3. Prior-mask normalization and projection

Because genes differ substantially in the number of linked peaks, raw prior weights can bias attention toward genes with many candidate regulatory elements. To reduce this bias, prior weights were normalized within each gene over peaks linked to the same gene using L1 normalization. Specifically, for gene g and linked peak p, the row-normalized prior weight $M_{g,p}^{\mathrm{norm}}$ is defined as:

$$M_{g,p} = \frac{\tilde{M}_{g,p}}{\sum_{p' \in \mathcal{N}_g} \tilde{M}_{g,p'}} \tag{1}$$

where $\tilde{M}_{g,p}$ represents the original confidence score (or binary indicator, $\tilde{M}_{g,p} \in \{0, 1\}$) for the gene–peak interaction, and $\mathcal{N}_g = \{p \mid \tilde{M}_{g,p} > 0\}$ denotes the set of candidate regulatory peaks associated with gene g.

Gene and peak features were encoded as low-dimensional tokens, and QK^T remained a gene-by-peak attention matrix compatible with the topology of the high-dimensional prior matrix M. The additive prior mask derived from M is injected into the attention logits before the softmax operation, with unsupported gene-peak pairs set to $-\infty$, so that unsupported gene-peak pairs receive zero attention mass. The model implementation keeps the mapping between gene-peak attention entries and original feature identifiers to allow downstream interpretation.

In Eq. (5) of the main text, $M_{g,p}$ denotes the normalized prior weight for a gene-peak pair. The dissimilarity term $dist(.,.)$ denotes the latent-space dissimilarity used for prior regularization between the projected gene embedding q_g and peak embedding k_p. In all experiments, $dist(q_g, k_p)$ was implemented as cosine distance. Embeddings were L2-normalized before computing this term.

4. Attention extraction and gene-peak scoring

After model training, normalized attention weights were extracted from the softmax output of the prior-guided cross-modal attention module. For multi-head attention, attention matrices were averaged across heads. When multiple cross-modal Transformer layers were used, the layers selected for interpretation were averaged consistently across all analyses.

The resulting attention matrix was mapped back to the original gene and peak identifiers using the feature dictionaries used during prior construction. For each gene, candidate peaks were ranked by attention-derived score. Top-k peaks per gene were used for link recovery, enrichment and locus-level visualization, with k specified in the corresponding figure legends or evaluation configuration. Cell-type-specific attention profiles were obtained by aggregating attention scores across cells assigned to the same cell type.

5. Peak-gene link recovery

To assess whether scHPGT attention recovers regulatory relationships, attention-derived gene-peak scores were compared with independent or held-out peak-gene links. Positive labels were defined by the selected reference link set, and negative labels were sampled from gene-peak pairs not present in the reference set while controlling for genomic distance when possible.

Performance was summarized using AUROC, Recall@K and enrichment fold. Enrichment fold was computed as the fraction of top-ranked attention links overlapping reference links divided by the corresponding fraction obtained from matched random links. Statistical significance was assessed by permutation testing in which gene-peak assignments were randomized while preserving the number of links per gene.

6. cCRE enrichment with matched background

High-attention peaks were intersected with ENCODE SCREEN candidate cis-regulatory element (cCRE) annotations. Enrichment was evaluated separately for promoter-like, proximal enhancer-like, distal enhancer-like, CTCF-bound and other regulatory categories.

To avoid confounding by genomic accessibility and peak properties, each high-attention peak set was compared with matched random background peaks. Background peaks were matched by chromosome, peak width, GC content and average accessibility whenever these covariates were available. Odds ratios were computed from 2×2 contingency tables and significance was assessed using Fisher's exact test and permutation testing.

7. Motif enrichment and cell-type specificity

For each cell type, top attention peaks were used for motif enrichment analysis. Motif enrichment was summarized using normalized enrichment scores (NES) and adjusted P values. To identify cell-type-specific regulatory programs, motif NES values were additionally normalized across cell types, producing differential motif-enrichment profiles that highlight lineage-biased transcription-factor programs.

Expected lineage markers include T cell-associated factors such as TCF7, LEF1, RUNX3 and TBX21; B cell-associated factors such as PAX5, EBF1 and SPIB; myeloid-associated factors such as SPI1, CEBPA and CEBPB; and NK/cytotoxic programs involving TBX21, EOMES, NKG7 and GNLY-associated regulatory modules.

8. Attention-derived regulatory activity

For gene g in cell c, scHPGT-derived regulatory activity was computed by weighting peak accessibility by attention-derived gene-peak scores:

$$R_{g,c} = \sum_{p \in P_g} A_{g,p} \times \mathrm{Accessibility}_{p,c}, \tag{2}$$

where P_g is the set of peaks linked to gene g and $A_{g,p}$ is the learned attention weight assigned to peak p for gene g. Regulatory activity was compared with matched gene expression and with known or inferred regulon programs. Correlations were computed across cells or metacells, and cell-type-specific activity matrices were visualized using z-score-normalized regulatory activity values.

9. Prior perturbation experiments

To test dependence on prior quality, we generated perturbed prior matrices. Conditions included the full prior, random removal of 20% of prior edges, random removal of 50% of prior edges, addition of 20% random edges, addition of 50% random edges, shuffled gene-peak assignments and a no-prior baseline. The shuffled-prior condition preserves matrix sparsity but destroys gene-peak specificity, and therefore tests whether incorrect biological structure can mislead the model.

Definitions of Noise and Perturbation Strategies:

- **Add $X\%$ Noise:** We introduced false-positive edges by randomly adding non-existent gene-peak connections equivalent to $X\%$ of the total edge count in the reference prior, simulating spurious regulatory priors.
- **Shuffled Prior:** We completely randomized all edge connections across the graph while preserving node identity and graph size, thereby completely destroying the biological mapping between peaks and target genes.

- **Drop $X\%$ Edges:** We randomly removed $X\%$ of the true biological edges from the original prior, simulating incomplete or missing regulatory annotations.

Each prior condition was evaluated using integration metrics (ARI, NMI, F1, BE and SI), activity–expression correlation and motif-enrichment NES. Robustness was interpreted as stable performance under moderate prior incompleteness or noise, with degradation under shuffled or absent priors indicating dependence on meaningful regulatory structure.

We further examined the prior with respect to three perturbation factors: the inclusion of distal edges, the enlargement of the distal window, and the replacement of binary values with correlation coefficients.

When the prior graph is restricted strictly to proximal edges, it exhibits the lowest overall metric performance among the newly tested configurations (Figure S12A, B), performing only slightly better than the complete absence of prior knowledge (No prior, Figure 8A). Proximal promoter regions primarily correspond to constitutively open chromatin that lacks strong cell-type specificity. Excluding distal enhancers strips the model of cell-type-specific regulatory signals that govern cell fate decisions. This severely diminishes cluster separability in the latent representation space, thus behaving similarly to the edge-deletion operation.

We also evaluated expanding the distal search window to 250 kb, 500 kb, and 1 Mb, respectively. At a 250 kb window, the evaluation metrics are slightly better than those of the Full prior, indicating that additional authentic cis-regulatory interactions are captured within the 150 kb–250 kb range. Expanding the window to 500 kb results in performance slightly inferior to the Full prior, while a 1 Mb window yields performance worse than the Drop 20% condition. This demonstrates that an excessively large search window leads to an exponential rise in false-positive genomic associations, injecting pseudo-noise edges into matrix M. This noise causes over-smoothing across distinct cell identities, ultimately reducing cell-type resolution.

We calculated co-accessibility scores to replace binary connections in matrix M, which achieved higher performance metrics than the Full prior. Compared to binary prior graphs, correlation-based continuous scoring provides superior feature representation quality and enhanced robustness against background noise. Therefore, constructing an appropriate continuous scoring scheme is a key direction for future model optimization.

10. Paired Data Integration Capability

We benchmarked the model on paired PBMC3k, paired PBMC10k, and the paired mouse brain SNARE-seq dataset (Figure S17). As shown in the results, scHPGT effectively aligns the two modalities while precisely resolving diverse cell types. In both PBMC3k-paired and PBMC10k-paired, scHPGT cleanly separated major cell lineages—such as B cells, macrophages, and T-cell subtypes. Similarly, on the SNARE-seq, it achieved clear segregation among distinct cortical cell types.

11. Statistical reporting

Unless otherwise stated, summary statistics are reported as mean or median values across genes, peaks, cell types or random perturbation replicates as appropriate. For permutation-based analyses, the number of permutations, random seed, matching criteria and multiple-testing correction procedure are provided in the code and figure legends. All biological validation analyses specify the genome build, gene annotation version and external annotation release used.

12. Integration metrics

We integrated scRNA-seq and scATAC-seq data, training a classifier to predict ATAC cell types. We evaluated the model using the following metrics: Adjusted Rand Index for clustering similarity, Normalized

Mutual Information for alignment with true labels, and F1 scores for a balanced measure of precision and recall. The Batch Entropy score to evaluate the extent of mixing cells across datasets and the Silhouette coefficient to evaluate the separation of biological distinctions.

- **Adjusted Rand Index** The Adjusted Rand Index (ARI) is a measure of the similarity between two clusterings, adjusted for chance. It ranges from -1 to 1, where 1 indicates perfect agreement, 0 indicates random labeling and negative values indicate worse-than-random agreement.

$$ARI = \frac{RI - ExpectedRI}{\max(RI) - ExpectedRI} \tag{3}$$

  where RI is the Rand Index, which measures the similarity between the clustering result and the ground truth labels. $ExpectedRI$ is the expected Rand Index under random label assignment.

- **Normalized Mutual Information** Normalized Mutual Information (NMI) quantifies the agreement between two clusterings, normalized to account for the number of clusters. It ranges from 0 to 1, where 1 indicates perfect agreement.

$$NMI = \frac{2 \cdot I(X, Y)}{H(X) + H(Y)} \tag{4}$$

  where $I(X, Y)$ is the mutual information between the clustering result X and the ground truth labels Y, while $H(X)$ and $H(Y)$ are the entropies of X and Y, respectively.

- **F1 Score** The $F1$ Score is the harmonic mean of precision and recall, commonly used to evaluate classification models, especially in cases of class imbalance. It ranges from 0 to 1, where 1 indicates perfect precision and recall.

$$F1 = \frac{2 \cdot TP}{2 \cdot TP + FP + FN} \tag{5}$$

  where TP (True Positives) are the correctly predicted positive samples, FP (False Positives) are the incorrectly predicted positive samples, and FN (False Negatives) are the positive samples incorrectly predicted as negative.

- **Batch Entropy** We computed the Batch Entropy (BE) score to quantifies the degree of mixing between cells from different datasets by evaluating the regional mixing entropies at the locations of randomly selected cells. A higher score indicates better integration and mixing of cells across datasets. The Batch Entropy score is computed using the following equations:

$$p_i^r = \frac{p_i/P_i}{\sum_{t=1}^{n} p_i/P_i}, \tag{6}$$

$$BE = -\sum_{t=1}^{n} p_i^r \log(p_i^r), \tag{7}$$

  where p_i represents the proportion of cells from batch i in a specific region, and P_i denotes the proportion of the total cell count that belongs to batch i.

- **Silhouette coefficient** The Silhouette Coefficient (SI) measures the compactness and separation of clusters. Its maximum value is 1, and the larger the value, the better the performance in distinguishing different clusters.

$$SI = \frac{b - a}{\max(a, b)} \tag{8}$$

where a is the average distance between a sample and all other samples in the same cluster, and b is the average distance between a sample and all samples in the nearest neighboring cluster.

13. Computational efficiency and resource allocation

We measured the wall-clock runtime and peak memory or video-memory usage of all benchmarking methods across datasets of varying scales. The detailed results are summarized in Table S2.

Table S2: Computational efficiency comparison across datasets of different scales.

Dataset	Metric	scHPGT	GLUE	uniPort	SCALEX	Cobolt	LIGER
PBMC3k	Runtime	5.8 min	9.5 min	6.8 min	2.2 min	4.8 min	13.5 min
	Peak memory	5.2 GB VRAM	4.5 GB VRAM	3.6 GB VRAM	1.8 GB VRAM	2.9 GB VRAM	6.8 GB RAM
Mouse spleen	Runtime	5.2 min	8.8 min	6.1 min	1.9 min	4.3 min	12.1 min
	Peak memory	4.8 GB VRAM	4.1 GB VRAM	3.3 GB VRAM	1.6 GB VRAM	2.6 GB VRAM	6.2 GB RAM
CITE & ASAP-seq	Runtime	6.2 min	10.2 min	7.4 min	2.5 min	5.2 min	15.2 min
	Peak memory	5.6 GB VRAM	4.9 GB VRAM	3.9 GB VRAM	2.0 GB VRAM	3.1 GB VRAM	7.6 GB RAM
Unbalanced-RNA	Runtime	15.5 min	22.4 min	16.8 min	5.4 min	13.8 min	41.2 min
	Peak memory	9.8 GB VRAM	11.2 GB VRAM	8.2 GB VRAM	3.8 GB VRAM	7.2 GB VRAM	19.8 GB RAM
Unbalanced-ATAC	Runtime	12.9 min	20.1 min	14.2 min	4.8 min	11.3 min	39.2 min
	Peak memory	7.5 GB VRAM	9.6 GB VRAM	7.4 GB VRAM	3.5 GB VRAM	6.5 GB VRAM	19.5 GB RAM

We systematically assessed computational efficiency using a standardized hardware setup. GPU-based methods were run on an NVIDIA RTX 4090 with 24 GB VRAM, while the CPU-based method was run on an Intel Xeon Gold 6430 system with 120 GB DDR4 RAM. All methods were evaluated using the computational resource configurations detailed in Table S3.

Table S3: Summary of computational resource allocation across methods.

Method	GPU/CPU	Accelerator	Memory	Parallelization	Computing unit
scHPGT	GPU	NVIDIA RTX 4090 (24 GB)	120 GB DDR4 RAM	CUDA v12.1	1× GPU (exclusive)
uniPort	GPU	NVIDIA RTX 4090 (24 GB)	120 GB DDR4 RAM	CUDA v12.1	1× GPU (exclusive)
GLUE	GPU	NVIDIA RTX 4090 (24 GB)	120 GB DDR4 RAM	CUDA v12.1	1× GPU (exclusive)
Cobolt	GPU	NVIDIA RTX 4090 (24 GB)	120 GB DDR4 RAM	CUDA v12.1	1× GPU (exclusive)
SCALEX	GPU	NVIDIA RTX 4090 (24 GB)	120 GB DDR4 RAM	CUDA v12.1	1× GPU (exclusive)
LIGER	CPU	–	120 GB DDR4 RAM	OpenMP (64 threads)	32 physical cores

Supplementary Figures

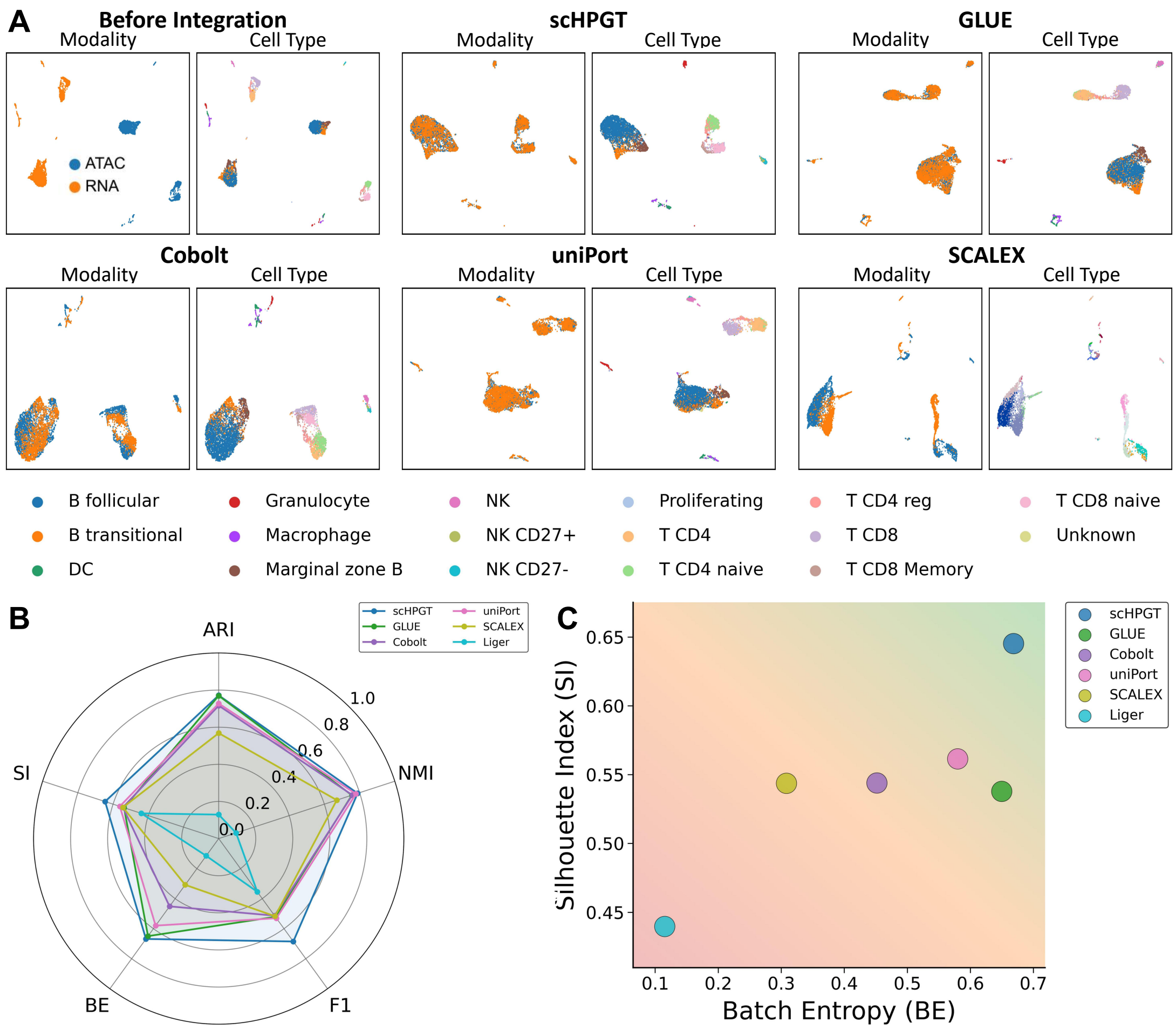

Figure S1: **scHPGT integrates unpaired scRNA-seq and scATAC-seq data from the Mouse spleen data while preserving the heterogeneity. (A)** UMAP embeddings of the raw data and integration results from different methods, colored by modality (RNA and ATAC) and cell type. **(B)** Performance comparison of each method across datasets, evaluated using ARI, NMI, F1 score, batch entropy (BE), and silhouette index (SI). **(C)** Trade-off between batch entropy (BE) and silhouette index (SI) across methods.

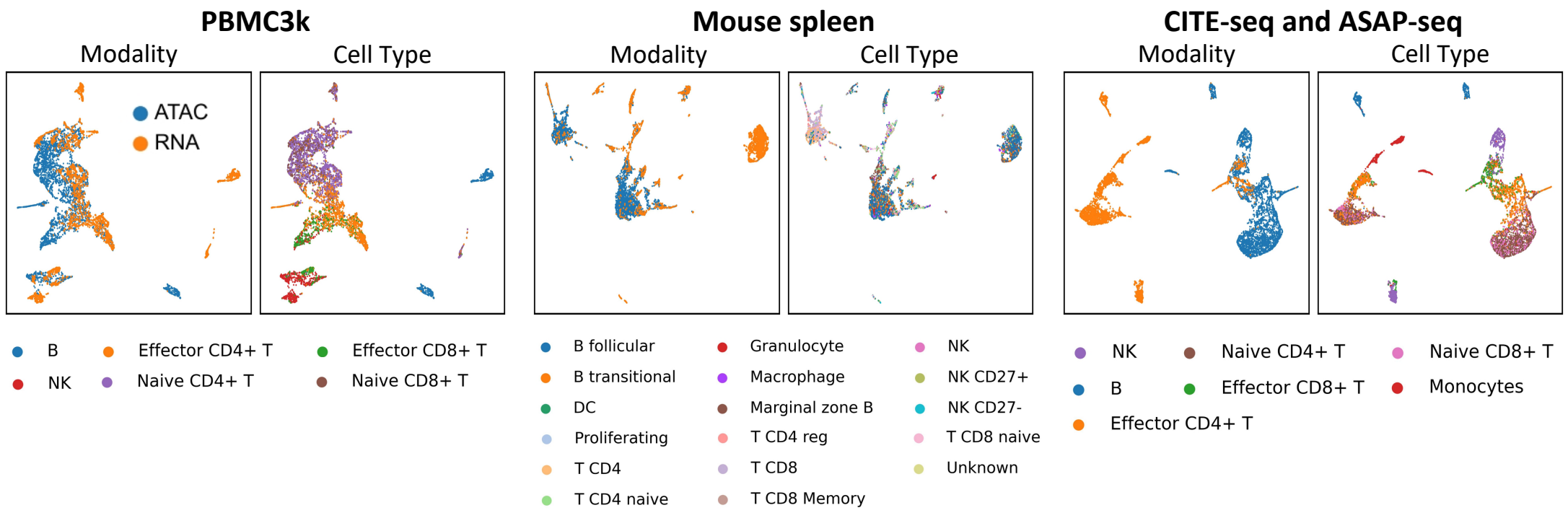

Figure S2: Visualization results of LIGER integrating PBMC3k, mouse spleen, and CITE-seq and ASAP-seq.

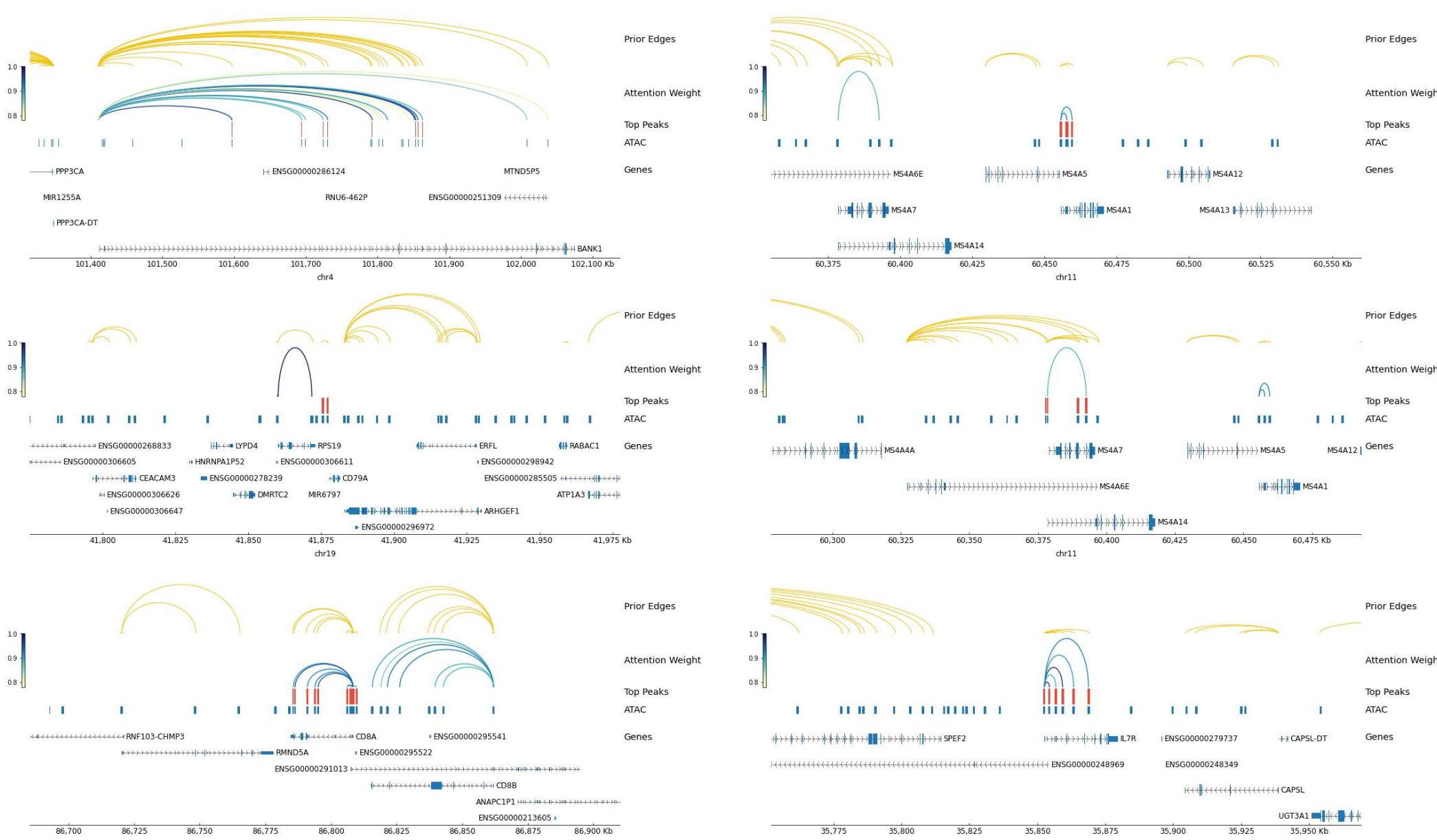

Figure S3: **Performance of Masked Attention on real gene–peak tracks.** The top panel shows the gene–peak connections in the prior graph. Top Peaks indicate peaks with high attention-based correlation with the target gene.

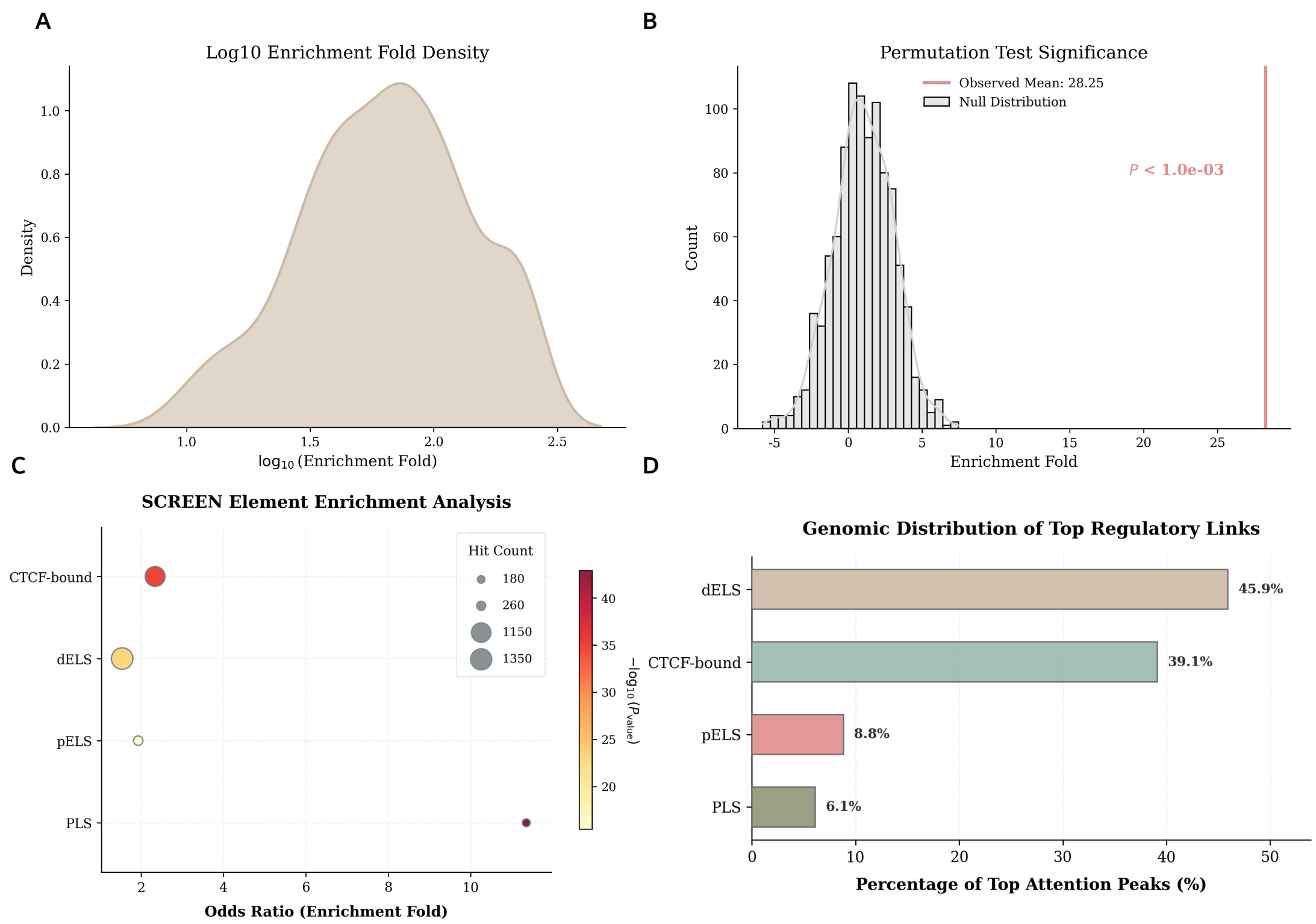

Figure S4: **Biological validation and functional annotation of high-attention peaks identified by scHPGT. (A)** Fold-enrichment scores of TF motifs in high-attention peaks. **(B)** Background motif fold-enrichment control based on randomized gene–peak associations. **(C)** Enrichment odds ratios and statistical significance for ENCODE candidate cis-regulatory element (cCRE) annotations. **(D)** Proportional distribution of functional cCRE classes among high-attention peaks.

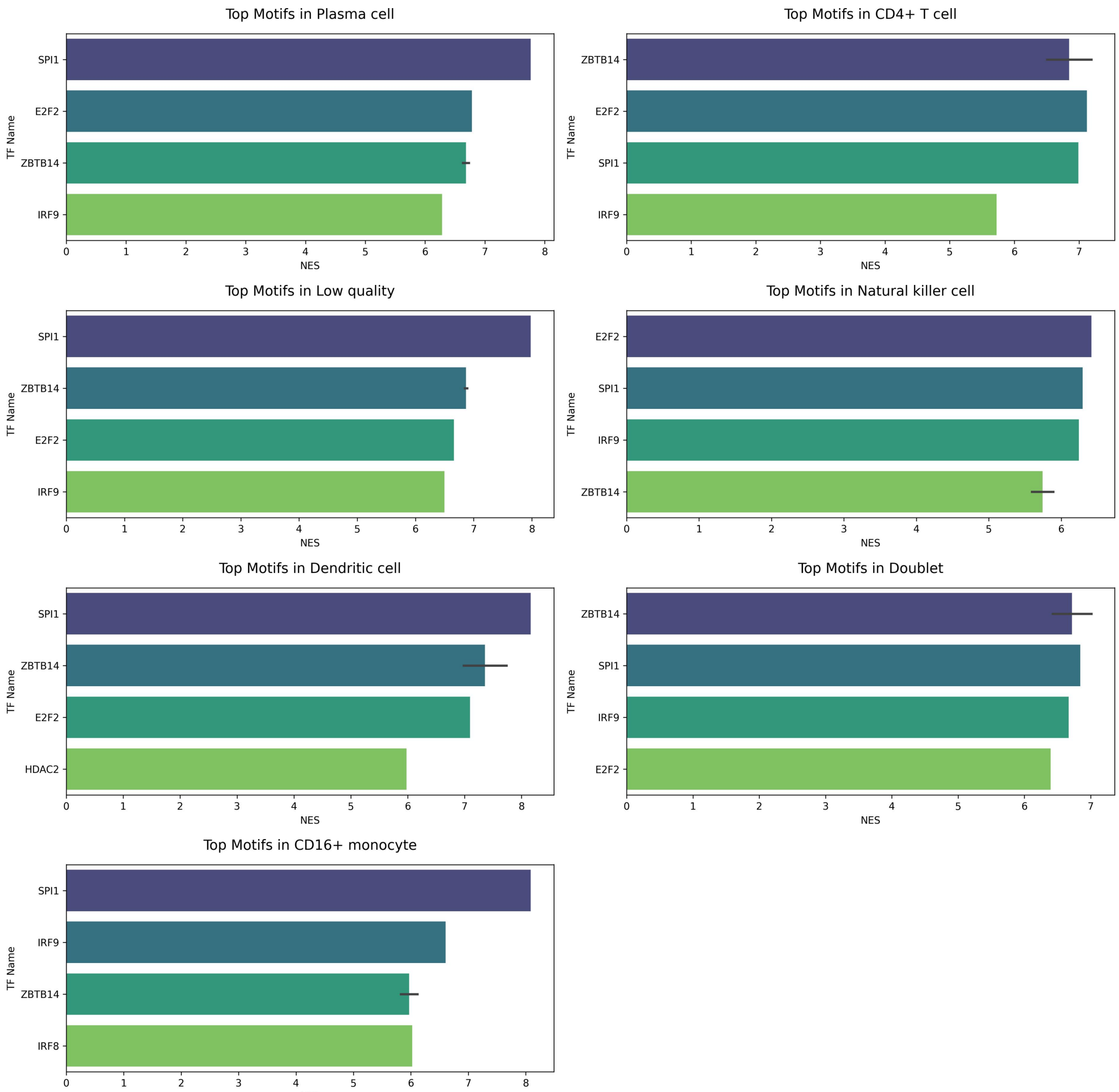

Figure S5: **Top transcription factors across different cell types.**

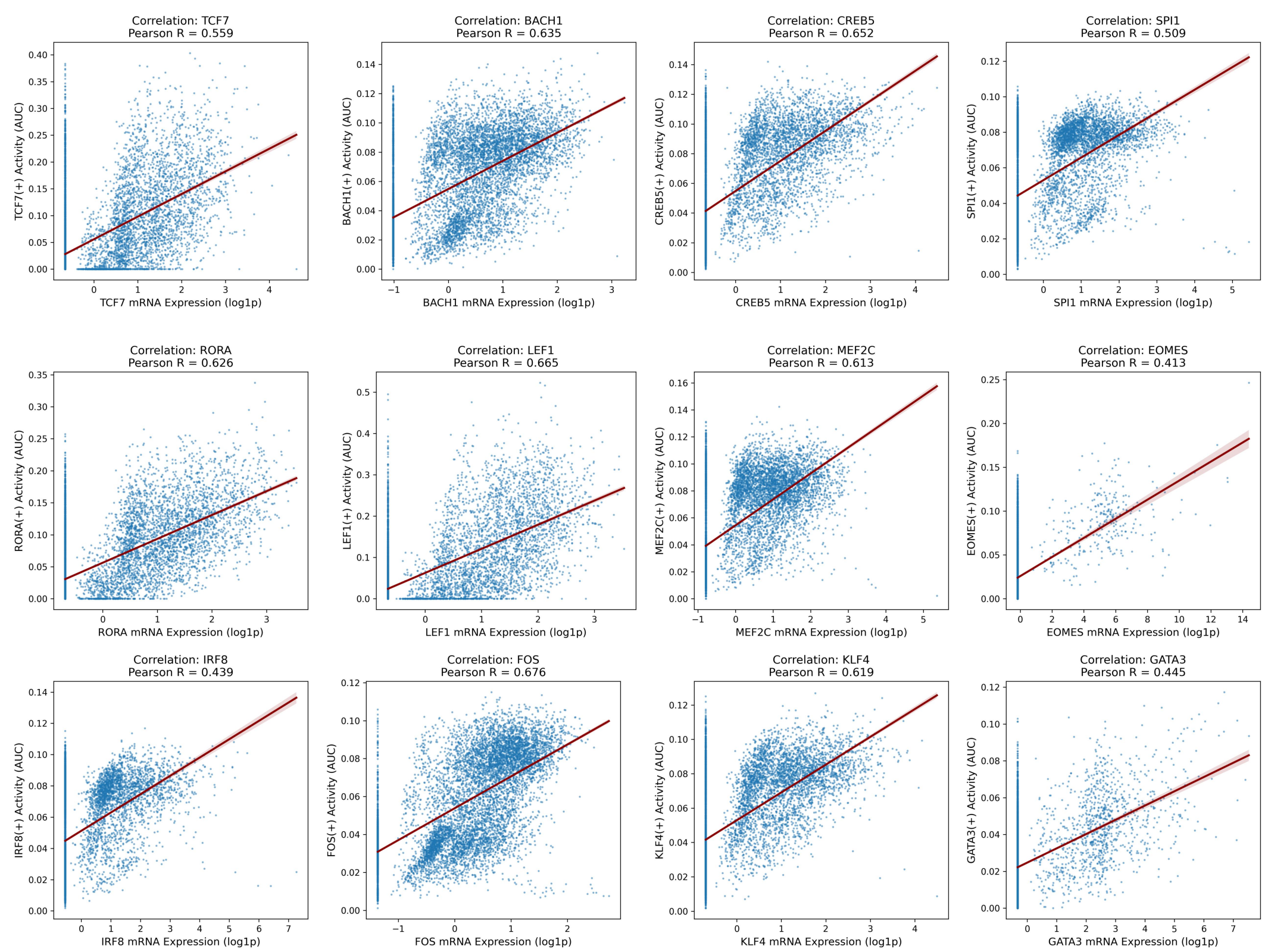

Figure S6: **Effects of gene expression levels on attention-derived regulatory activity across different marker genes.**

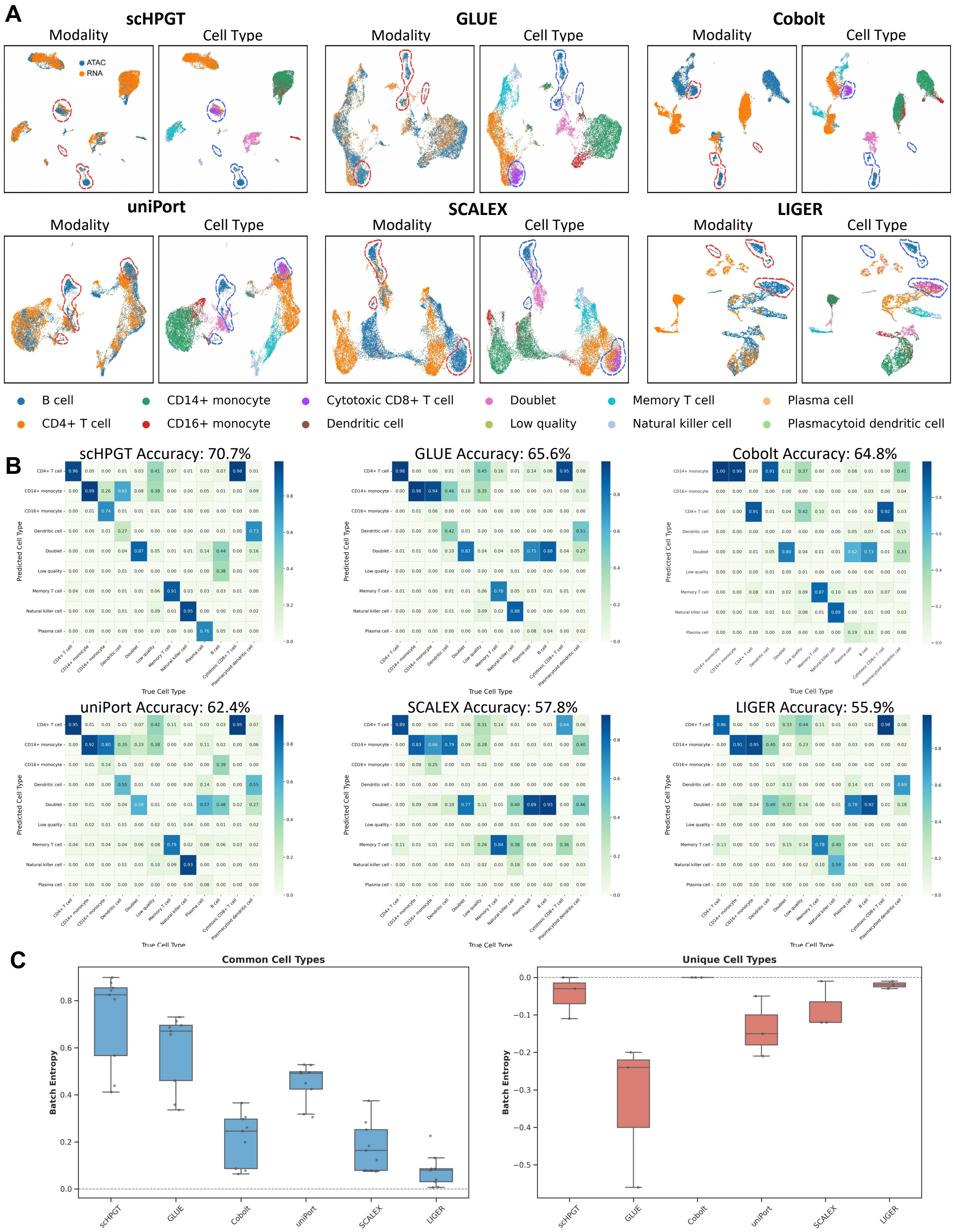

Figure S7: **Integration results under the Unbalance-RNA setting.** **(A)** UMAP embeddings under the Unbalance-RNA setting, colored by modality and cell type, with clusters of unique cell types highlighted. **(B)** Heatmap comparing original labels and transferred labels, with the average label-transfer accuracy of each method shown at the top. **(C)** Batch entropy of shared and unique groups. Left: shared-group results, where higher values indicate better alignment; right: unique-group results, where values closer to 0 indicate better separation of independent cell types.

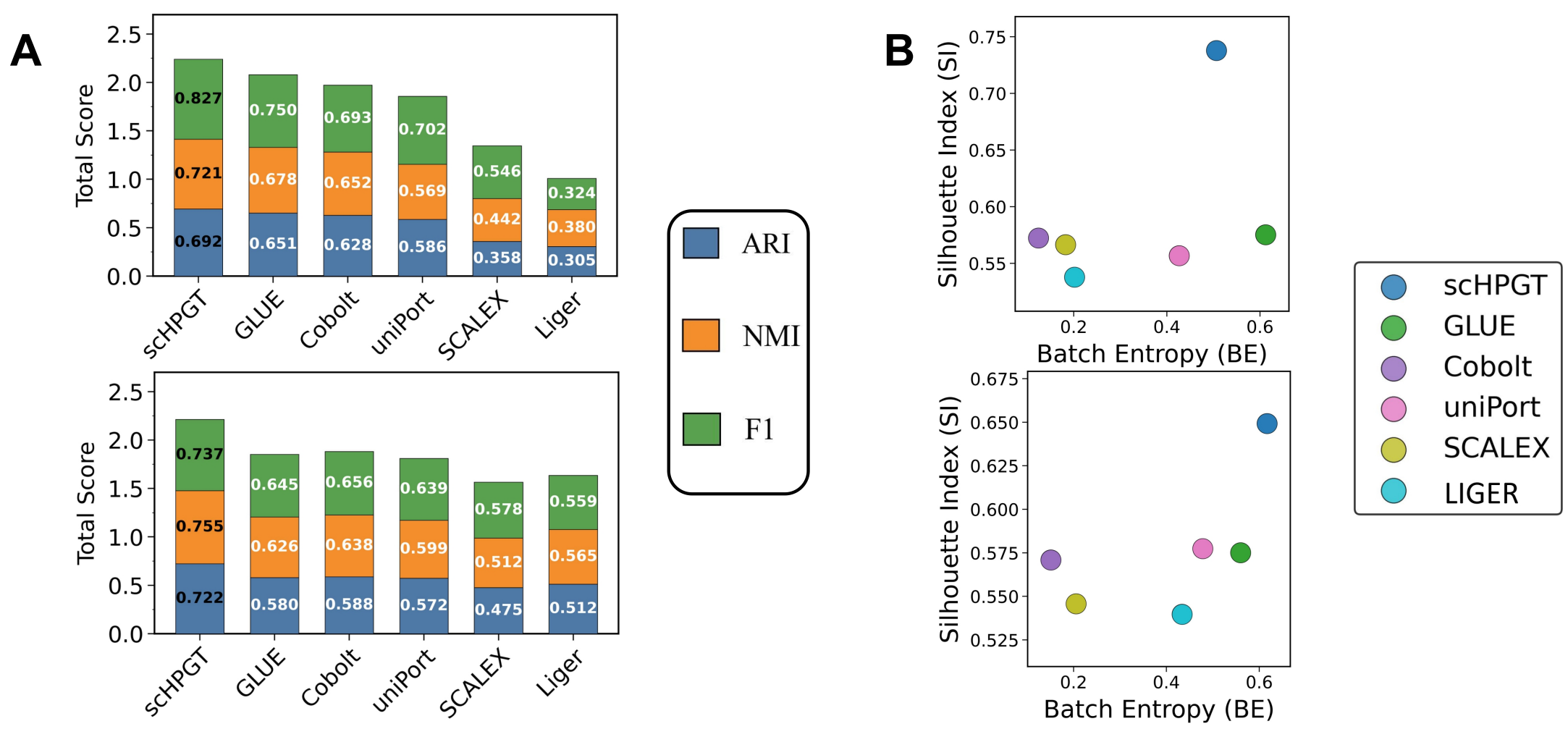

Figure S8: **Integration metrics of the Unbalance-RNA and Unbalance-ATAC settings.** **(A)** ARI, NMI, and F1-score comparison across methods (top: Unbalance-RNA; bottom: Unbalance-ATAC). **(B)** Batch Entropy (BE) and Silhouette Index (SI) comparison (top: Unbalance-RNA; bottom: Unbalance-ATAC).

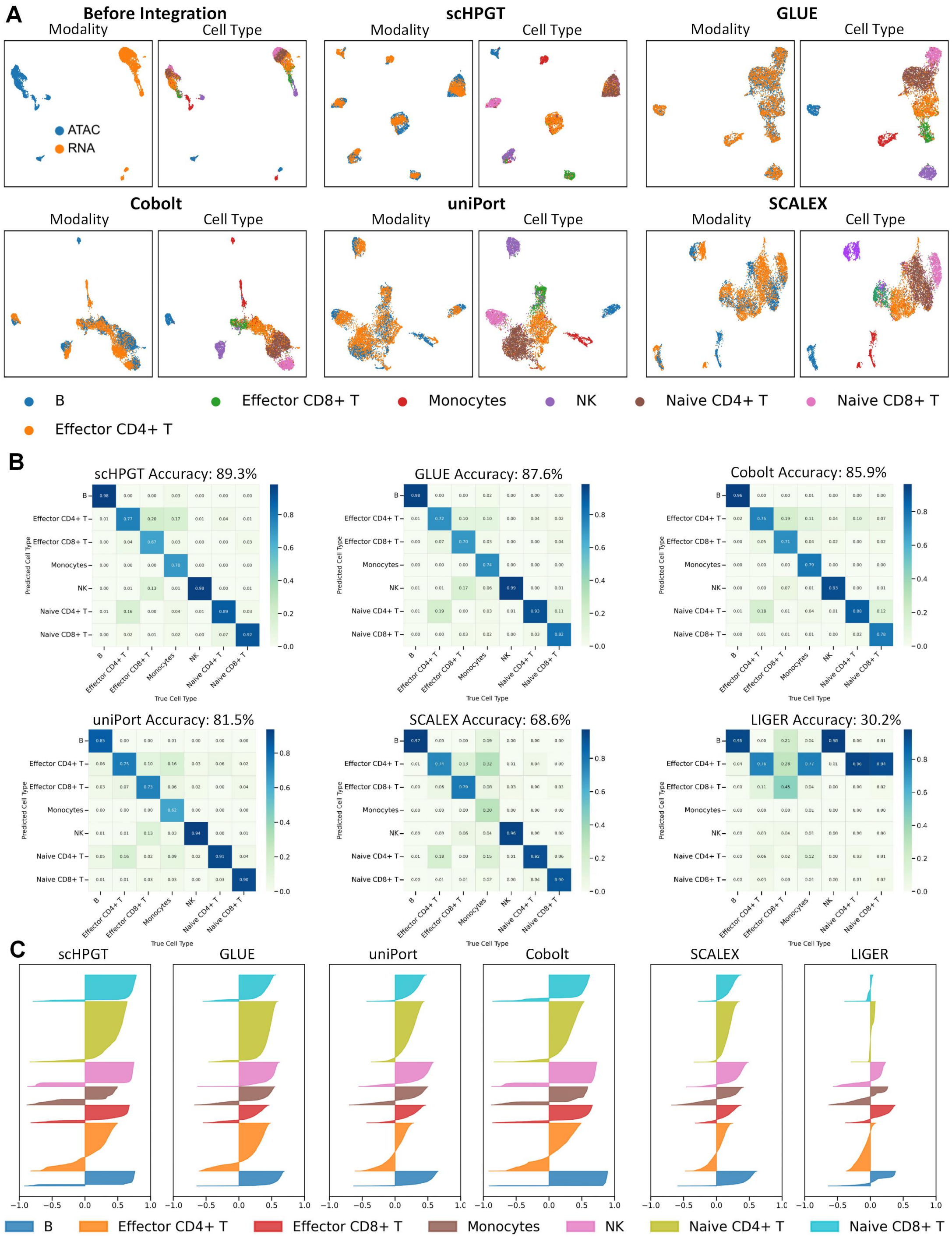

Figure S9: **Integration of multimodal PBMC data across biological conditions. (A)** UMAP embeddings of raw data and integration results, colored by modality (RNA/ATAC) and cell type. **(B)** Heatmap comparing original labels and transferred labels. **(C)** Cell-type silhouette coefficients across methods, where higher values indicate better grouping.

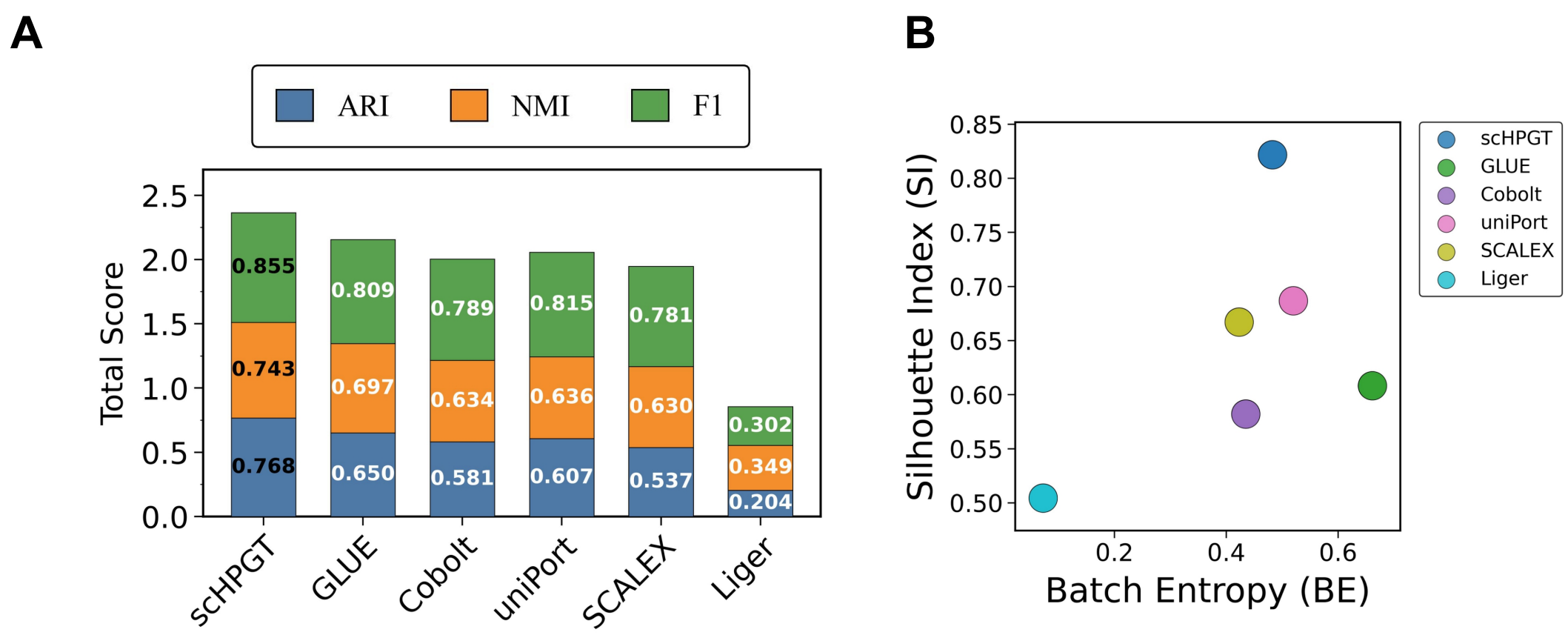

Figure S10: **Integration metrics of multimodal PBMC data across biological conditions. (A)** Performance comparison across methods and datasets using ARI, NMI, and F1 scores. **(B)** Trade-off between Batch Entropy (BE) and Silhouette Index (SI) across methods.

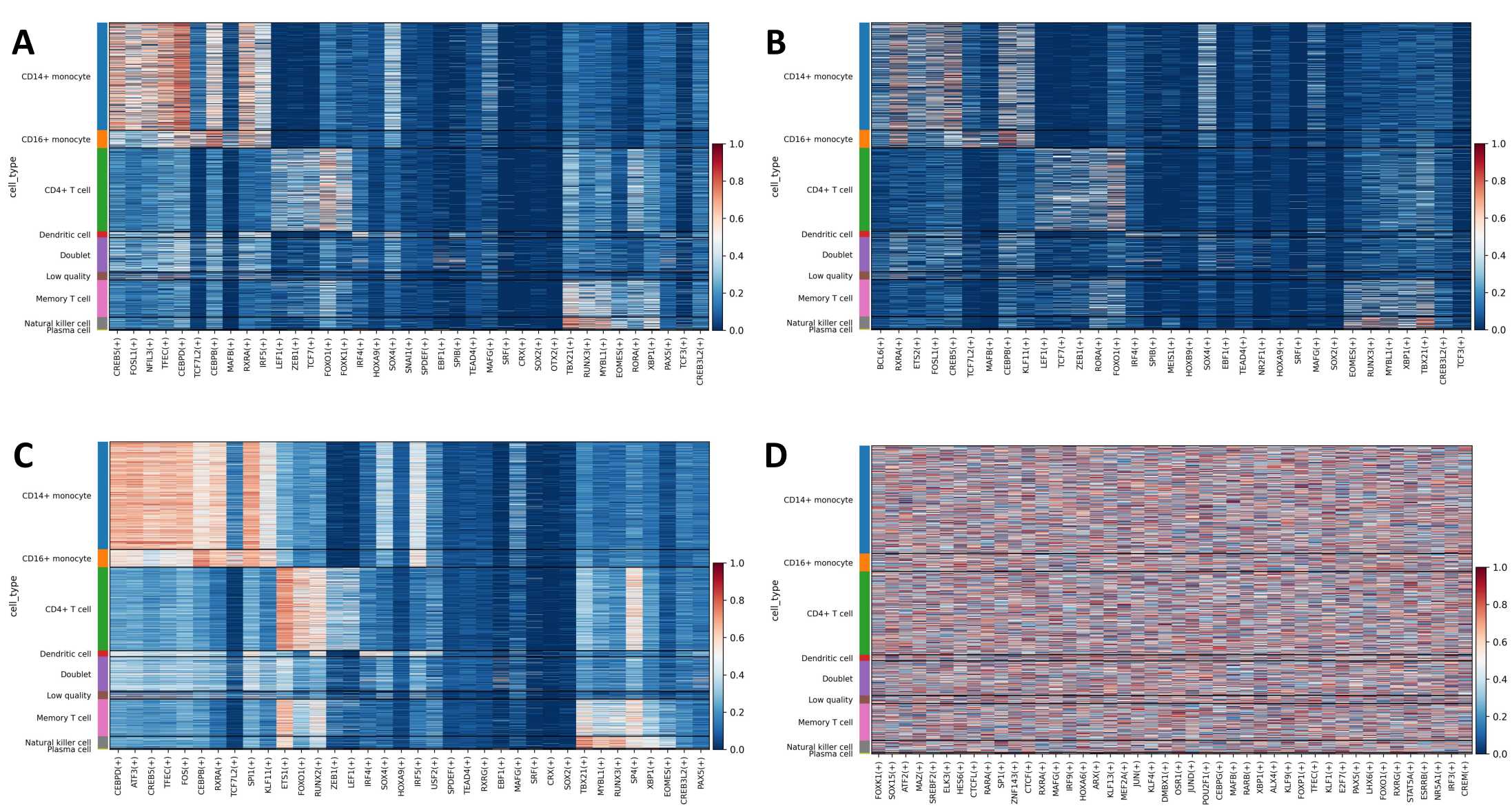

Figure S11: **Motif enrichment NES inferred from attention under different prior perturbation settings. (A)** Drop 20% edges. **(B)** Drop 50% edges. **(C)** Add 20% noise. **(D)** No prior.

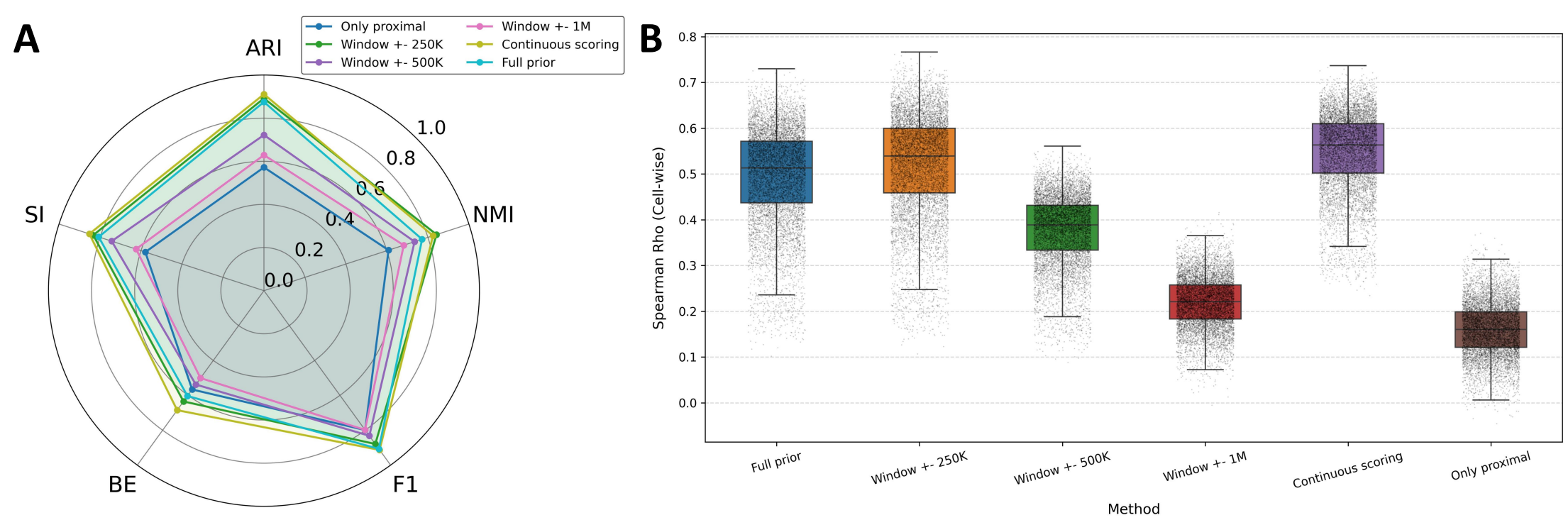

Figure S12: **Impact of prior graph spatial constraints on integration performance. (A)** Radar plots comparing integration metrics across different prior configurations. **(B)** Boxplots showing the distribution of Spearman correlation between attention-derived regulatory activity and gene expression under spatial constraints.

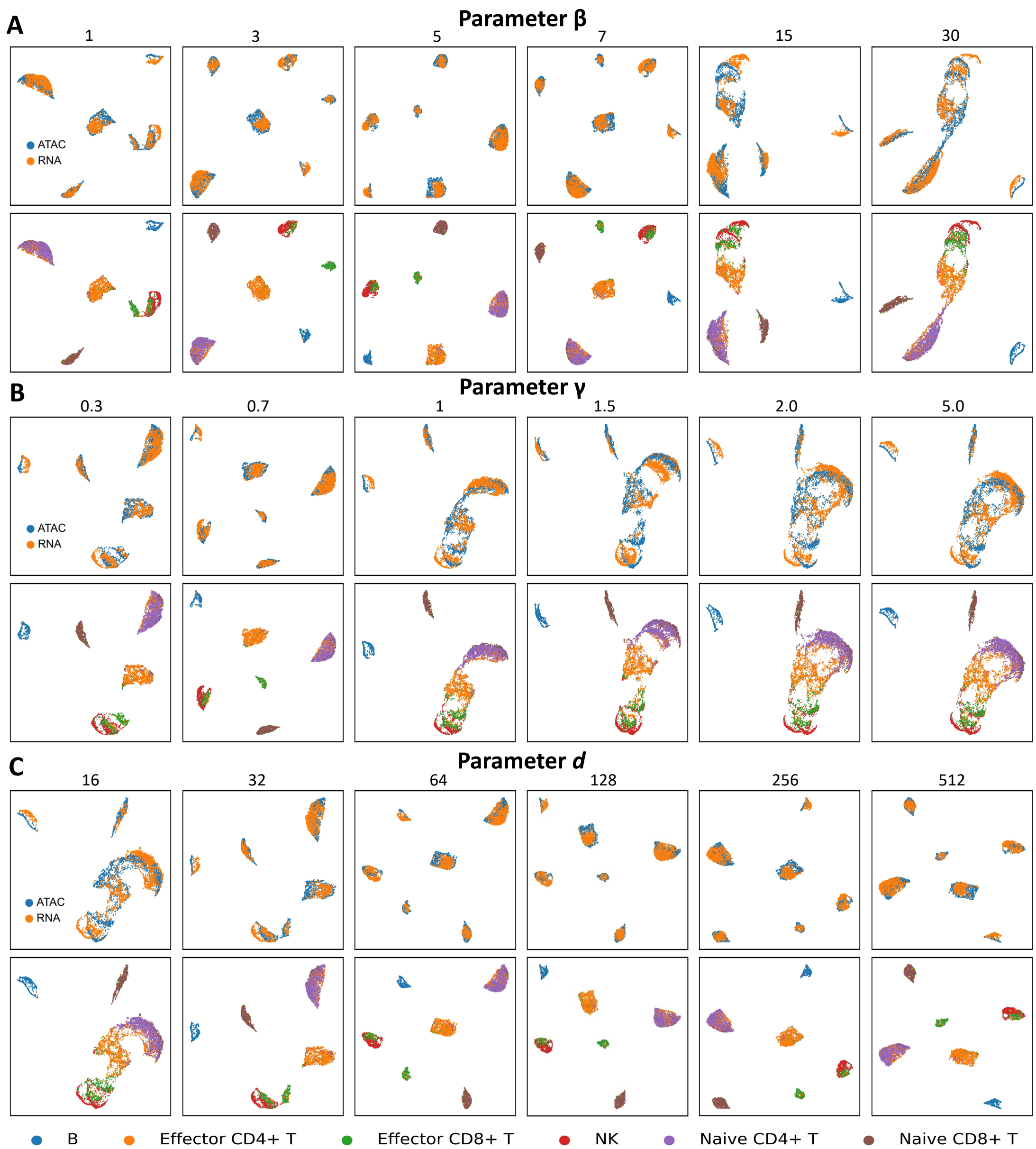

Figure S13: **Performance of scHPGT under different parameter settings in PBMC3k. (A)** UMAP embeddings with fixed γ and varying β. **(B)** UMAP embeddings with fixed β and varying γ. **(C)** UMAP embeddings across different values of d. For each setting, modality distributions are shown above cell-type distributions, with cell-type labels at the bottom.

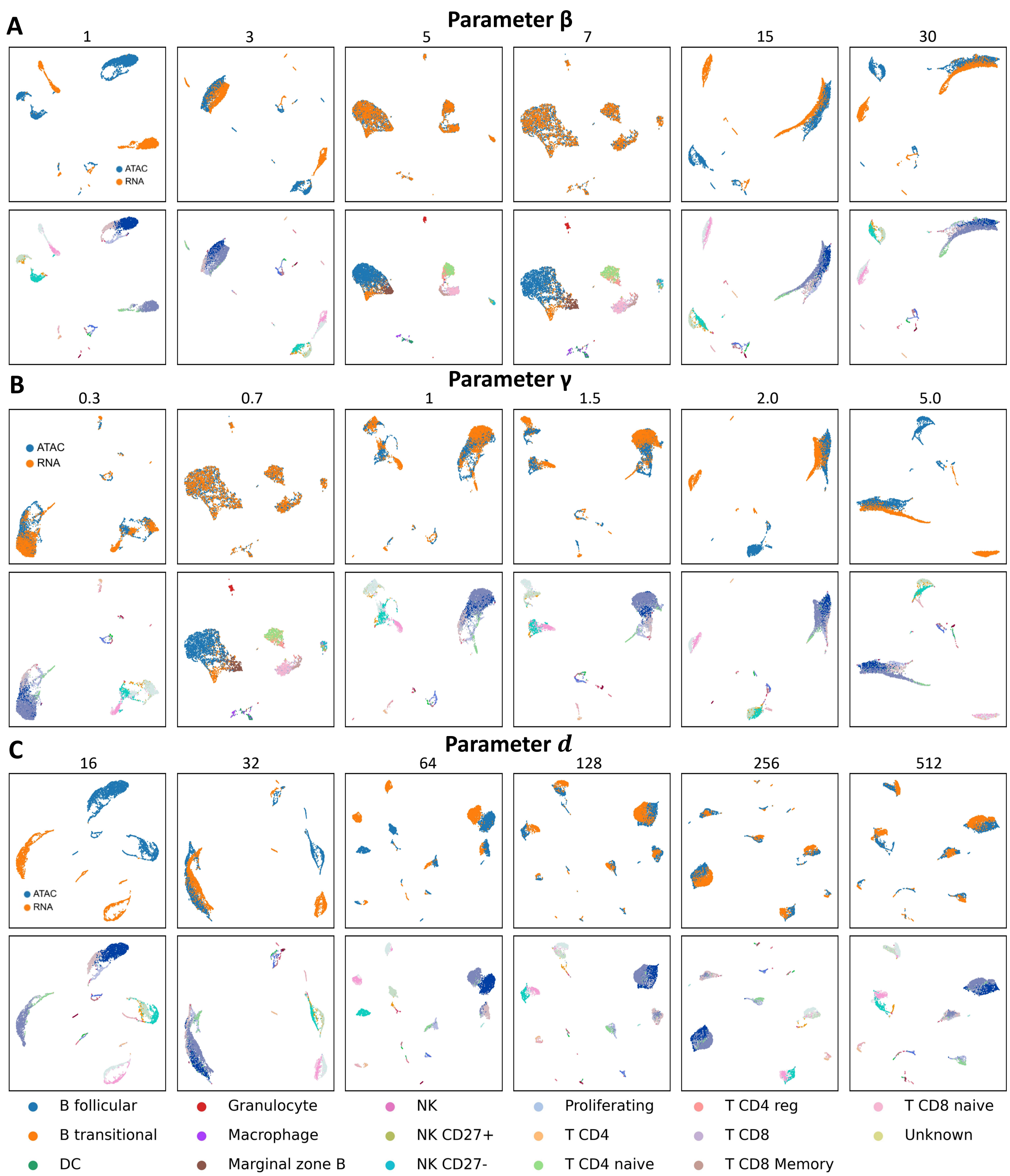

Figure S14: **Performance of scHPGT with varying parameters across Mouse spleen.** **(A)** UMAP embeddings with fixed γ and varying β. **(B)** UMAP embeddings with fixed β and varying γ. **(C)** UMAP embeddings across different values of d. For each setting, the first row shows modality distributions and the second row shows cell-type distributions. The bottom shows cell-type labels.

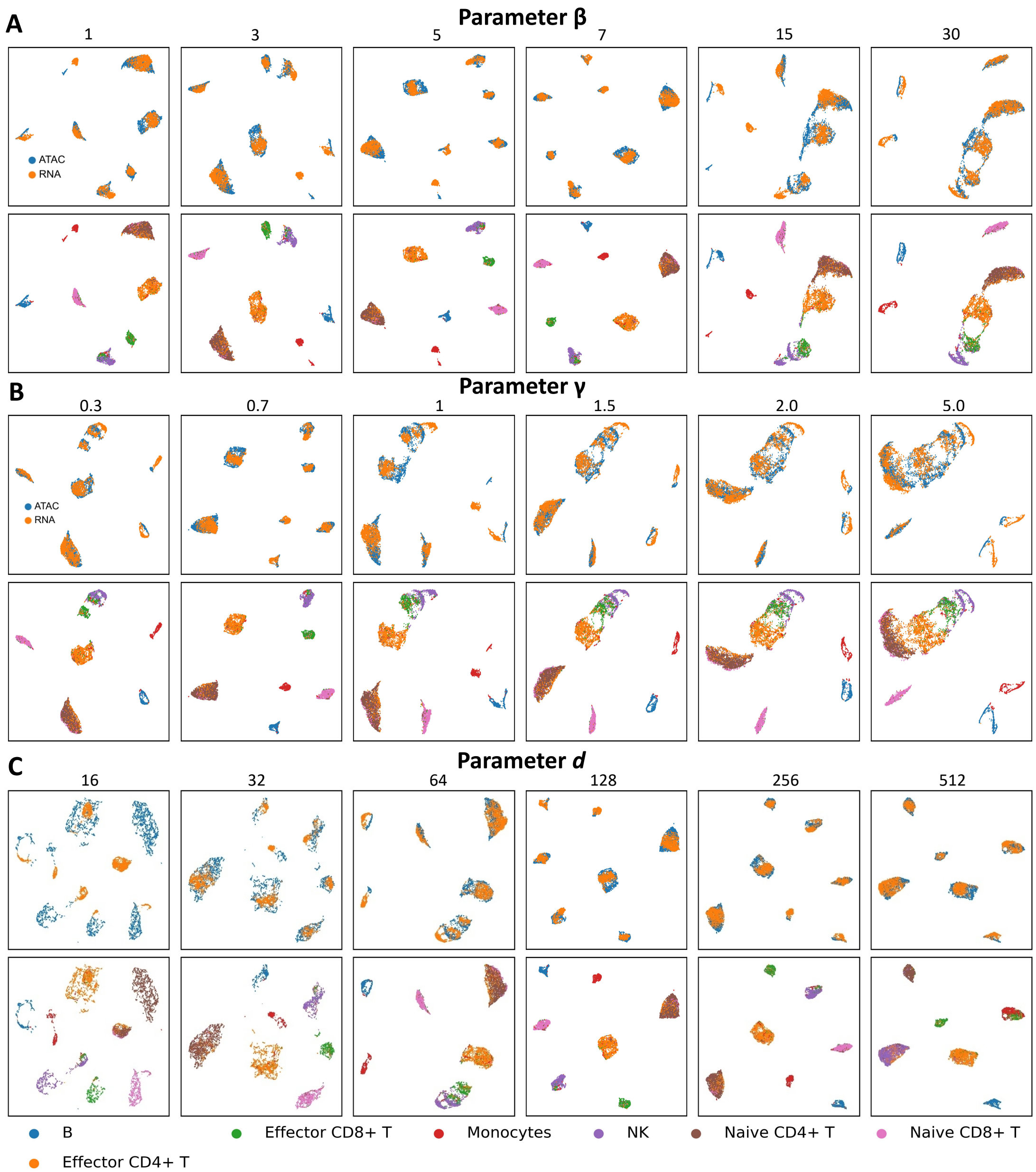

Figure S15: **Performance of scHPGT with varying parameters across CITE-seq and ASAP-seq PBMC.** **(A)** UMAP embeddings with fixed γ and varying β. **(B)** UMAP embeddings with fixed β and varying γ. **(C)** UMAP embeddings across different values of d. For each setting, the first row shows modality distributions and the second row shows cell-type distributions. The bottom shows cell-type labels.

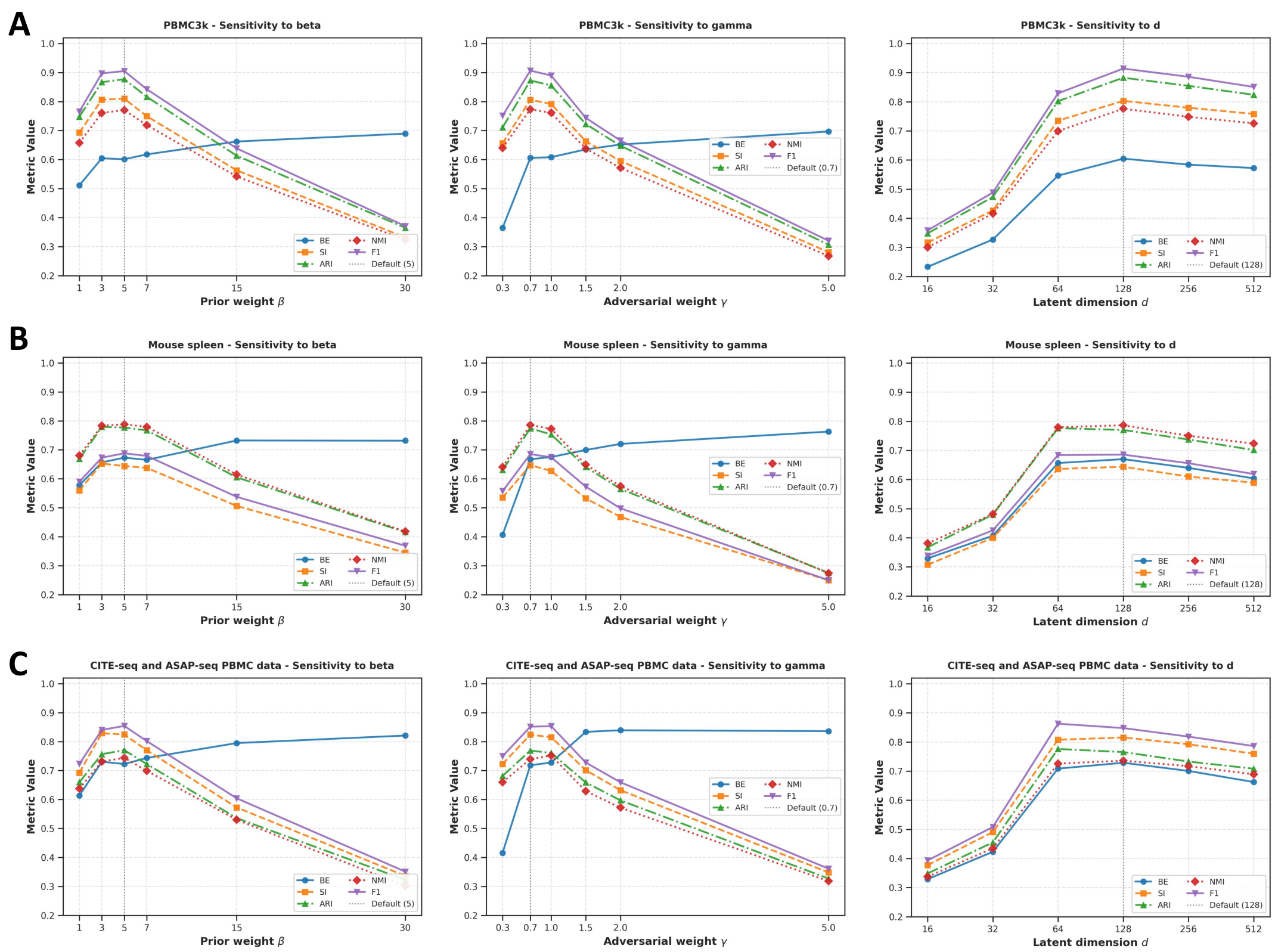

Figure S16: **Parameter sensitivity analysis of scHPGT across benchmark datasets.** Evaluation of five performance metrics under varying hyperparameter configurations (β, γ, and d) for **(A)** PBMC3k, **(B)** CITE-seq & ASAP-seq PBMC data, and **(C)** Mouse spleen. Each panel displays sensitivity curves for prior weight β (left), adversarial weight γ (middle), and latent dimension d (right), with vertical dotted lines marking default settings.

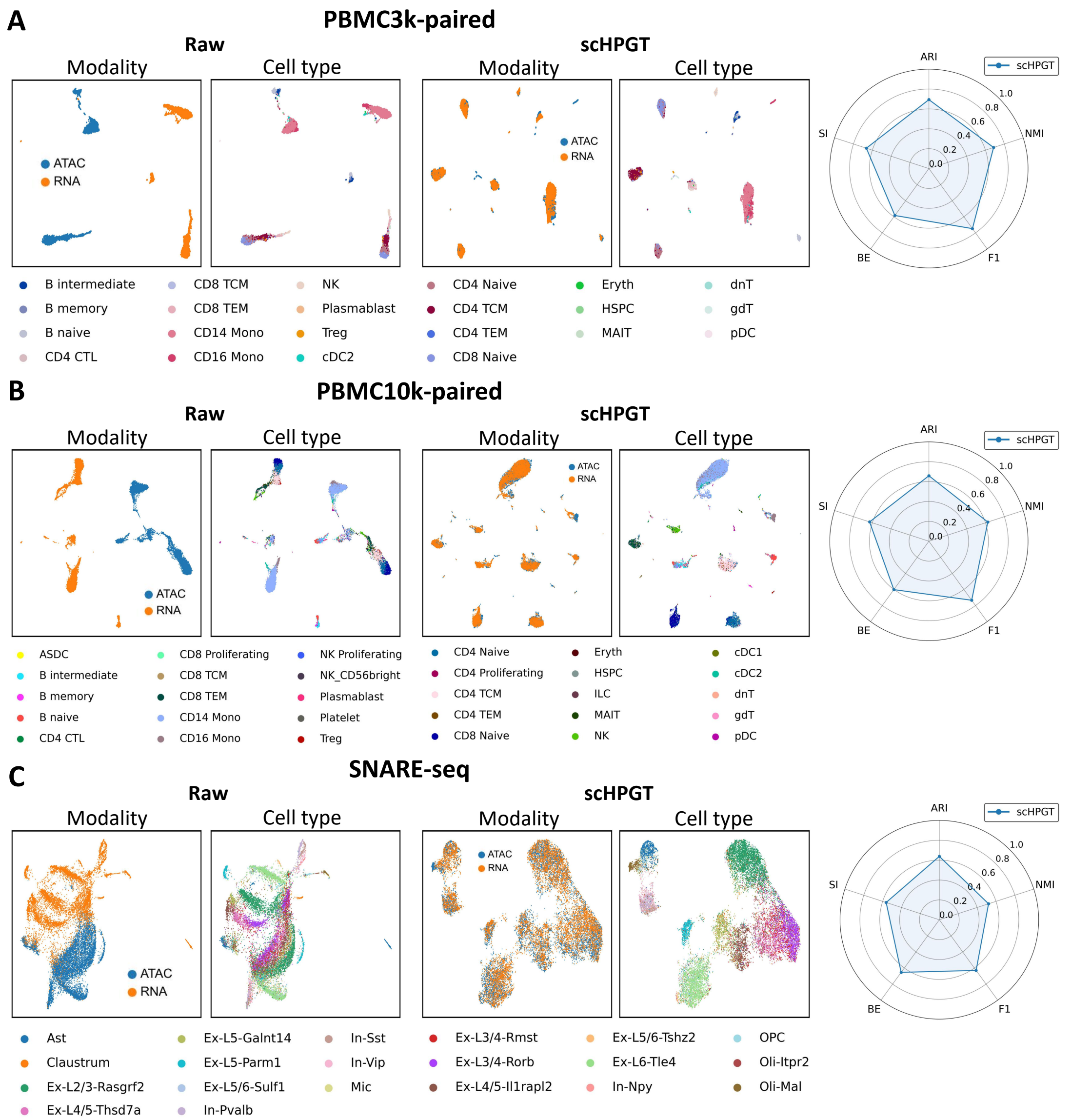

Figure S17: **Integration performance and benchmark evaluations of scHPGT across paired multimodal datasets. (A–C)** Unintegrated (left) and scHPGT-integrated (middle) UMAP visualizations, alongside quantitative benchmark metrics (right), across three paired multimodal datasets: **(A)** PBMC3k-paired, **(B)** PBMC10k-paired, and **(C)** SNARE-seq.
